# Electrotaxis of Glioblastoma and Medulloblastoma Spheroidal Aggregates

**DOI:** 10.1038/s41598-019-41505-6

**Published:** 2019-03-29

**Authors:** Johnathan G. Lyon, Sheridan L. Carroll, Nassir Mokarram, Ravi V. Bellamkonda

**Affiliations:** 1 Department of Biomedical Engineering, Pratt School of Engineering, Duke University, 101 Science Drive, Durham, NC 27705 USA; 2 Wallace H. Coulter Department of Biomedical Engineering, Georgia Institute of Technology & Emory School of Medicine, UA Whitaker Building, 313 Ferst Drive, Atlanta, GA 30332 USA

## Abstract

Treatment of neuroepithelial cancers remains a daunting clinical challenge, particularly due to an inability to address rampant invasion deep into eloquent regions of the brain. Given the lack of access, and the dispersed nature of brain tumor cells, we explore the possibility of electric fields inducing directed tumor cell migration. In this study we investigate the properties of populations of brain cancer undergoing electrotaxis, a phenomenon whereby cells are directed to migrate under control of an electrical field. We investigate two cell lines for glioblastoma and medulloblastoma (U87mg & DAOY, respectively), plated as spheroidal aggregates in Matrigel-filled electrotaxis channels, and report opposing electrotactic responses. To further understand electrotactic migration of tumor cells, we performed RNA-sequencing for pathway discovery to identify signaling that is differentially affected by the exposure of direct-current electrical fields. Further, using selective pharmacological inhibition assays, focused on the PI3K/mTOR/AKT signaling axis, we validate whether there is a causal relationship to electrotaxis and these mechanisms of action. We find that U87 mg electrotaxis is abolished under pharmacological inhibition of PI3Kγ, mTOR, AKT and ErbB2 signaling, whereas DAOY cell electrotaxis was not attenuated by these or other pathways evaluated.

## Introduction

Primary brain and nervous system cancers represent only a small proportion (1.4%) of total cancer incidence, however, these cancers are involved in proportionally twice as many cancer mortalities (2.8%) with a mere 65% 5-year survival rate^1^. Persons with the most common pediatric brain malignancy, medulloblastoma, fare only slightly better than average, with a 71.9% 5-year survival, though the most common adult brain tumor, glioblastoma multiforme (GBM), incurs a mere 5.1% 5-year survival rate^2^. These dismal survival rates are due in part to a lack of available therapies for cancers in neural tissues, as the brain is a particularly difficult organ to treat. The brain has several eloquent, inoperable regions, and these cancers often disseminate and disperse deep into neural tissue, forming micro-satellites that make surgical resection difficult and often impossible^3,4^. Even if resection is possible, recurrence is common, due to an inability to completely resect invasive fronts penetrating beyond the primary tumor core^5,6^. Chemotherapy is also hindered in the brain, as transport is restricted by physiological barriers to the blood stream^7^. Even with continual advances in drug discovery and delivery technologies that have substantially improved outcomes in systemic cancers, there has been disappointingly little impact on tumors of the brain. Thus, there is an urgent need to innovate new strategies to safely manage or treat brain cancers and therein a substantial need for a better understanding of how brain tumor dissemination can be limited or controlled. Here, we look to electrical fields and their influence on cellular behavior as a means to control tumor dispersion, using the regime of low field-strength direct current, wherein electrotaxis occurs.

Electrotaxis (sometimes referred to as galvanotaxis), is the phenomenon whereby application of a low voltage, direct current electrical field (dcEF) provides a cue to direct a cell to move of its own volition^8,9^. The concept of a non-contact force being able to direct a cell’s motility is potentially interesting for brain cancer therapy when you consider that controlling tumor dispersion–or possibly undoing it–could be a major boon to the management of brain tumors.

Electrotaxis has been observed as an endogenous response to physiologically-generated electric fields (3–500 V/m) occuring during wound healing, neural development, and cancer invasion, as part of the ensemble of numerous, simultaneous, chemical and physical cues in each process^10–14^. Upon application of a dcEF, the phenomenon has been observed in myriad cell types, over a range of fields strengths, leading to a variety of cell-type specific responses that as of yet have no generalizeable, unifying mechanism^15,16^. Applied fields in this range have been studied extensively^15^, and have broadly shown no negative impact on cell viability (as the fields are outside of the range that would disrupt cell membranes or produce joule heating^17^) and thus may be useful in a therapeutic strategy to differentially impact cells that exhibit electrotaxis from those that do not. This also makes the strategy distinct from another recent cancer electrotherapy, tumor-treating fields^18^, whereby, an alternating EF is used and the effect on cell viability is frequency dependent and requires fields that alternate in the 100–300 kHz range.

For cancer research, electrotaxis was first explored as a way to characterize the metastatic potential of cells^19^, with further research showing the trend of more metastatic cells having stronger electrotactic response toward a cathode (the negative source electrode)^20–22^ and in some cases, weakly metastatic cells of the same ilk, having a diminished response in the opposite direction relative to the applied field, i.e., toward an anode (the positive source electrode).

Brain cancers, however, have been studied in only a few recent electrotaxis studies, with a focus mainly on mechanistic studies. 2-dimensional (2D), sparsely plated human glioblastoma multi-forme (GBM) cells (U251 and U87 mg) and C6 rat glioma cells have been found to electrotax toward the cathode and stimulated the production of hydrogen peroxide and superoxide^23,24^. Recently, Huang, *et al*., showed that the directional effect may be further altered by substrate or plating conditions. After observing a variety of brain-tumor-initiating cells with an anodal electrotactic preference on 2D substrates, these same cells were plated in a 3-dimensional (3D) matrix of hyaluronic acid and collagen, wherein the electrotactic response of the brain-tumor-initiating cells reversed toward the cathode, but the authors presented no further evidence as to why this had occurred^25^.

It remains unclear, given any particular cell, whether it will electrotax, or in which direction it will move, and given the startling finding that substrate or plating conditions may dramatically alter the electrotactic response, there is a need for more studies and electrotactic platforms that expand beyond the traditional, short-term, 2D, single cell format.

### Establishment of a population-density, 3-dimensional, electrotaxis system using brain cancer spheroidal aggregates

Much of the previous work on electrotaxis has been focused on single-cell responses on 2D substrates, however, when cancerous tissues grow or invade in tissues, they may also do so as a collective or in 3D^26^. There have been few studies of electrotaxis at population or tissue densities. Babona-Pilipos, *et al*. studied neurospheres of subependymal neural precursors plated on a 2D substrate, but only analysed cells that had migrated sufficiently far from the rest of the neurosphere cohort^27^. Cohen, *et al*., developed methods to track individual cells in epithelial sheets of arbitrary geometries, as a way to piece apart the varied electrotactic responses of leader and follower cells^28^. Lalli & Asthagiri showed, using densely-packed 2D cultures of mammary epithelial cells, that collective migration was more sensitive to dcEF (responding to 50% weaker fields than dispersed cells), yet the resulting electrotactic alignment occurred with slower dynamics^29^. One limitation to most population studies is that few cells truly form populations in 2D.

3D electrotaxis studies have thus far been limited due to inherent technical difficulties, but there have been a couple attempts of note. Zhang, *et al*., showed that dispersed human induced-pluripotent stem cells that were typically stationary in a 3D matrix, responded cathodally to a physiological-level dcEF^30^. Zhao, *et al*., developed a clever, high-throughput method of assaying cells in arrays of 3D agarose droplets with Dictyostelium cells, though this method has yet to be attempted for mammalian cell culture^31^. Spheroids have also been used as a 3D format though the studies have not included a 3D substrate and the spheroids have not been assessed for collective migration^27,32^. Most recently, the above-mentioned study by Huang, *et al*., on brain-tumor initiating cells that had differing electrotactic responses in 3D and 2D, highlights the need for more studies that consider experiments more than just 2D, single cell assays^25^.

**Figure 1:**
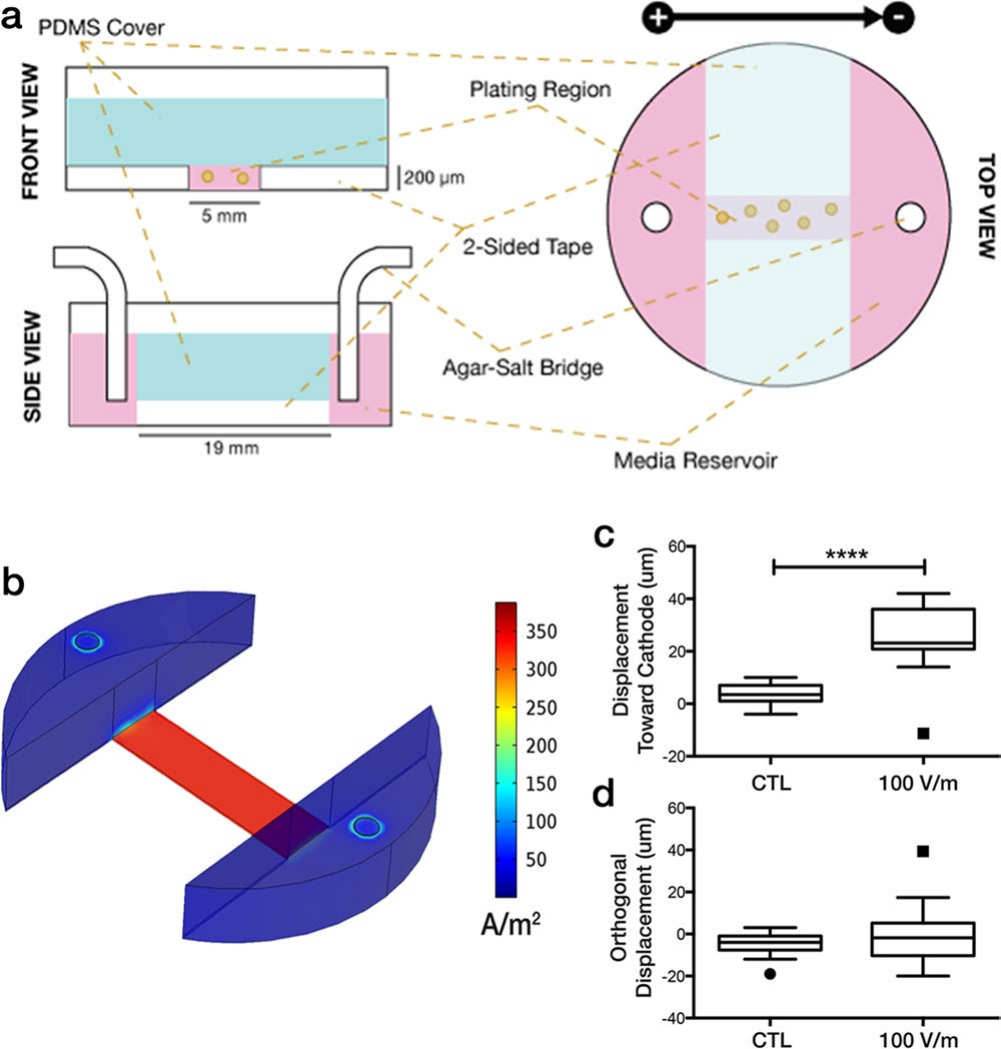
Design of our electrotaxis system. (**a**) Schematic of electrotaxis channel construction. (**b**) Computation validation using COMSOL with I_set_ = 330 μA (**c**,**d**) Biological validation using MatLyLu single cell tracking. Cells were tracked over 2 h for both no electrical field (CTL, n = 20) and under dcEF equivalent to 100 V/m (n = 22) for (**c**) displacement along the electrical field lines toward the cathode; and (**d**) displacement along the axis orthogonal to the electrical field. ****p < 0.0001 by Student**’**s t-test, two-tailed.

To provide a platform for 3D electrotaxis, we have attempted to marry the standard tumor invasion assay^33^ with previous work on standard electrotaxis assays^34^. The design principles we chose to build for were: (i) flexible configuration, to allow many potential plating options; (ii) simplicity in fabrication; (iii) capability for long-term (>4 h) experiments; and (iv) ability to analyze population-level changes without live-cell microscopy. We developed a system designed from standard 6-well tissue culture plates, replaceable salt bridges, and simple dcEF sources to allow for constant incubation (Fig. 1a). Apart from the single-cell tracking experiments, we developed a facile analytical method to allow for analysis of population behavior without the need for live-cell imaging.

Our custom electrotaxis chambers were validated computationally, using COMSOL Multiphysics, to verify that a consistent current density would be applied within the plating channel (Fig. 1b). Subsequently, we were able to reproduce previous results using the MatLyLu cell line that is known to electrotax toward a cathode (Fig. 1c,d)^19^. We were able to show results similar to those found in Djamgoz, *et al*.: after 2 h of exposure to a 100 V/m electrical field, MatLyLu cells moved over twice as much as unexposed cells. The control cells also had only a small net displacement (3.95 ± 3.86 μm) compared to exposed cells that were significantly more attracted to the cathode (26.11 ± 11.75 μm) (Fig. 1c). Comparison of the net field-orthogonal displacement, which should not be affected by an electrical field, resulted in no significant difference (Fig. 1d). *Note: the particular methods and dimensions we used are detailed in the methods section, however, these can be readily adjusted, as long as the cross-sectional geometry of the plating channel is consistent, and the channel is long enough to establish a region of consistent current density (verified by COMSOL modeling in our case)*.

We next used this platform to explore the electrotactic behavior of brain cancer cells in 3D. Using the Aggrewell method we produced spheroidal aggregates for embedding in growth-factor reduced Matrigel. Images of the aggregates were collected over time and used to measure the outmost growth of each of their four frontiers aligned to the electrical field: cathodal, anodal, orthogonal and orthogonal’ (Supplementary Fig. S1). Using these frontiers, we also defined the cathodal bias (the difference of cathodal to anodal frontier growth) and as control, an orthogonal bias (the difference of the orthogonal to orthogonal’ frontier growth).

**Figure 2:**
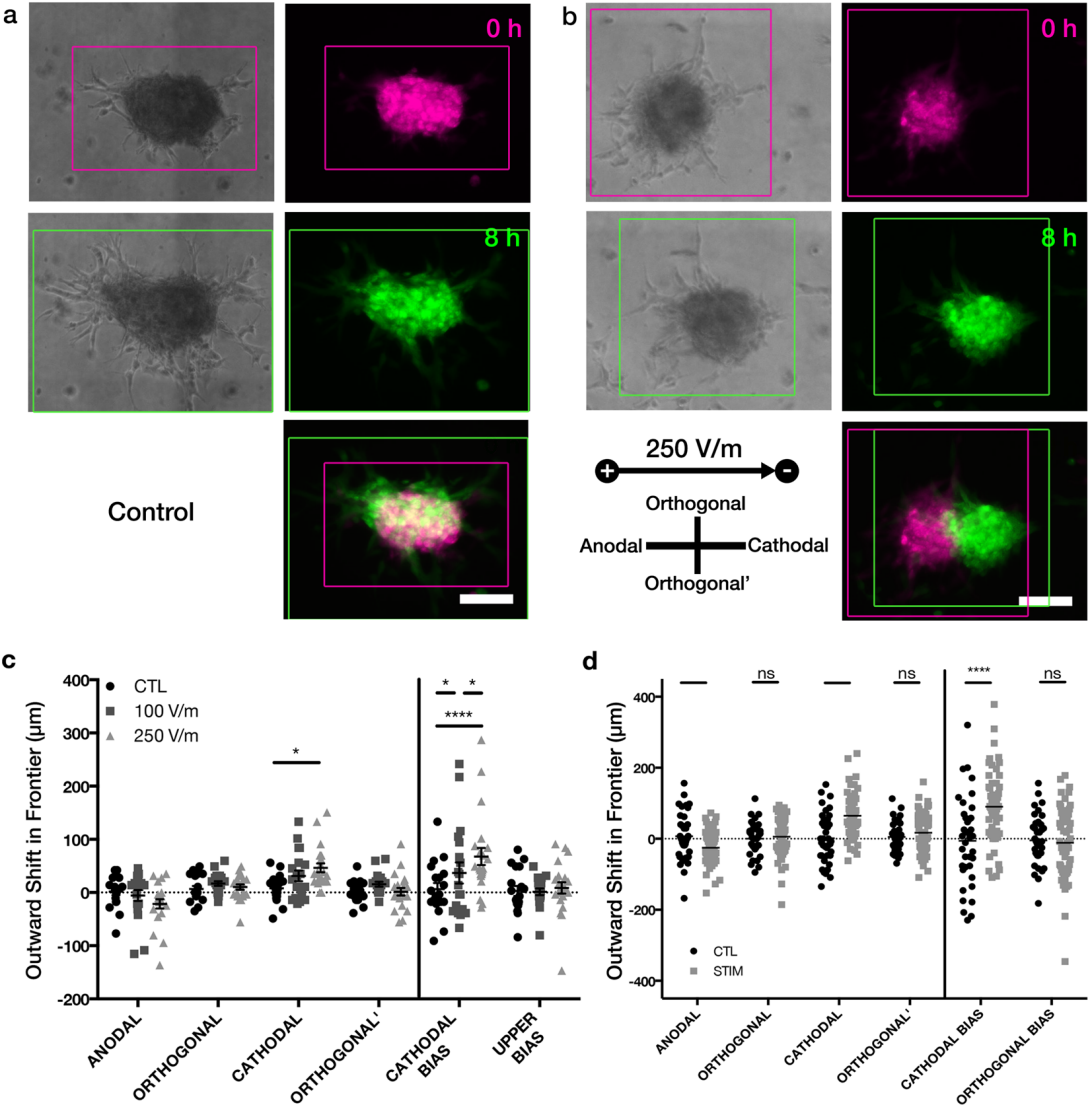
U87 mg spheroidal aggregates exhibit cathodal electrotaxis. (**a**) GFP + U87 mg control (no dcEF) aggregates showing no bias in growth direction after 8 h. (**b**) GFP + U87 mg aggregates exposed to 250 V/m dcEF exhibiting cathodal bias, detectable within 8 h. (**a**,**b**) are pseudo-colored for time = 0 (pink), time = 8 h (green). (**c**) Quantitative assessment of the outward shift of aggregate frontiers after 8 h for no electrical field (CTL, n = 19), 100 V/m (n = 19), and 250 V/m (n = 22). For cathodal frontier: *p = 0.0142; for cathodal bias: *p = 0.0448, ****p < 0.0001; by Two-way ANOVA and Holm-Sidak post-hoc test. Mean ± SEM shown. (**d**) Quantitative assessment of the outward shift of aggregate frontiers after 24 h for U87 mg with no dcEF (CTL, n = 39) or 250 V/m (STIM, n = 56). **p = 0.0032; ***p = 0.002; ****p < 0.0001; by Two-way ANOVA and Holm-Sidak post-hoc test. Scale: 150 μm.

For our 3D electrotaxis screening, we used human U87 mg cells, a widely used model of glioblastoma that has been previously studied for 2D electrotaxis^23,25^. Under an 8 h dcEF, our 3D U87 mg aggregates yielded a significant difference for both the cathodal frontier (for 250 V/m dcEF relative to control, p = 0.0142) and the overall cathodal bias (for both 100 and 250 V/m dcEF conditions relative to control, p = 0.0448 and p < 0.0001 respectively) (Fig. 2a–c) showing a mean cathodal bias of 68 ± 16 μm for 250 V/m, for 100 V/m, 37 ± 20 μm and 6 ± 12 μm for controls (mean ± s.e.m.). The cathodal bias for 100 V/m and 250 V/m were also found to be significantly field-strength-dependent and different for the two applied fields (p = 0.0448). The anodal frontier was not significantly different, though the mean response did decrease with increased voltage. However, what the conservative bounding box method does not capture is that the bulk of the aggregate has shifted while leaving behind trailing pseudopodia or immobile cells that are included when we demarcate frontiers. Extending the dcEF exposure to 24 h also maintains the cathodal response, with the aggregates exhibiting a significant difference in both cathodal frontier shift (p = 0.0002) and cathodal bias (p < 0.0001) relative to controls (Fig. 2d).

**Figure 3:**
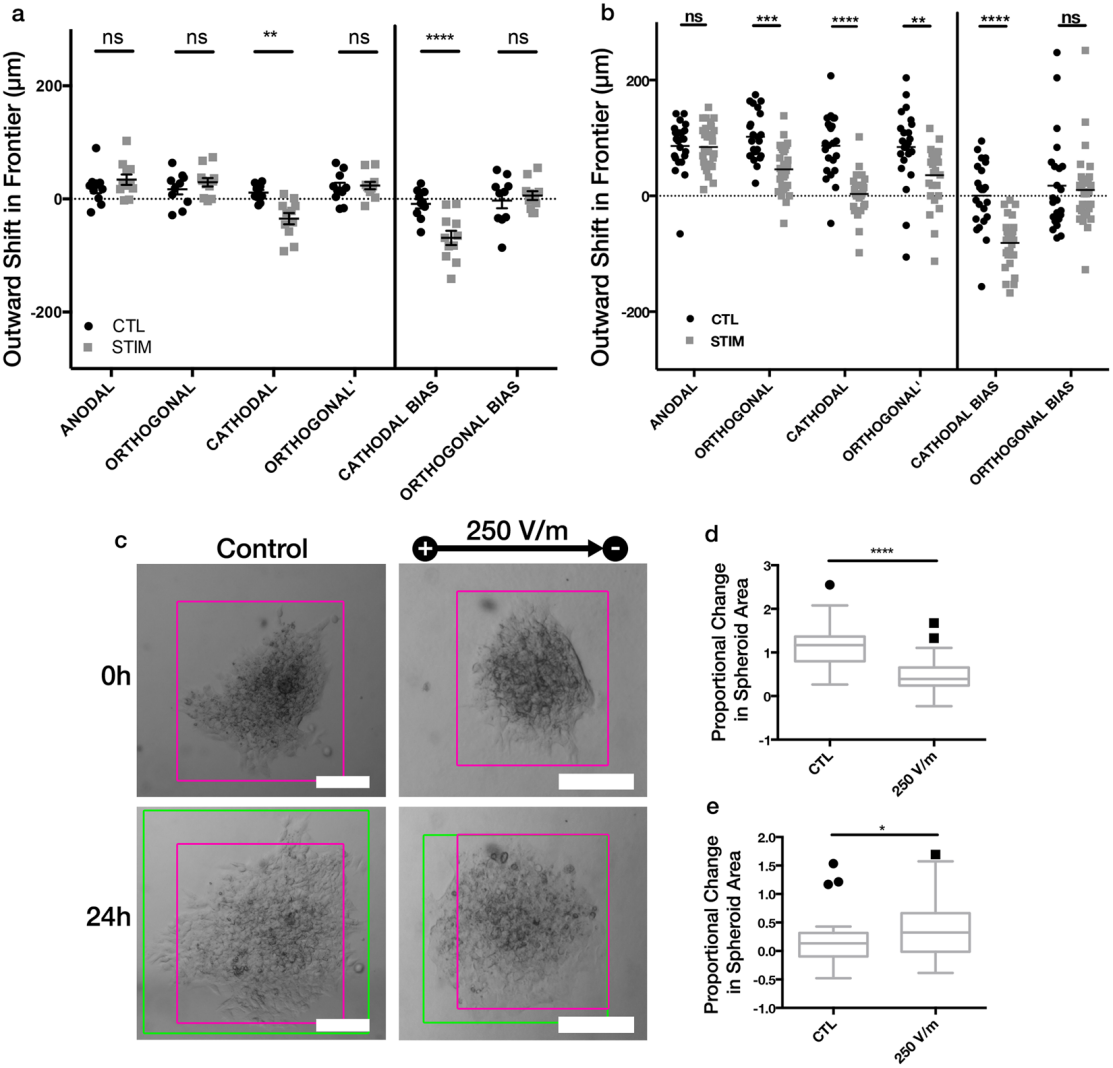
DAOY spheroidal aggregates exhibit anodal electrotaxis. (**a**) Quantitative assessment of the outward shift of aggregate frontiers after 8 h for no electrical field (CTL, n = 10) and 250 V/m (STIM, n = 11). **p = 0.0032; ****p < 0.0001; by Two-way ANOVA and Holm-Sidak post-hoc test. Mean ± SEM shown. (**b**) Quantitative assessment of the outward shift of aggregate frontiers after 24 h for DAOY with no dcEF (CTL, n = 23) or 250 V/m (STIM, n = 29). **p = 0.0032; ***p = 0.0007; ****p < 0.0001; by Two-way ANOVA and Holm-Sidak post-hoc test. (**c**) Example spheroid morphology before and after experiment, with and without 250 V/m dcEF for DAOY spheroidal aggregates after 24 h. Bounding boxes are colored for time = 0 (pink), time = 24 h (green). (**d**,**e**) Proportional change in spheroid area from time = 0 to time = 8 for (**d**) DAOY, and (**e**) U87 mg spheroidal aggregates. *p = 0.0133; ****p < 0.0001; by One-way ANOVA and Holm-Sidak post-hoc test. Mean ± Tukey box plots shown. Scale: 200 μm.

We next applied dcEFs to spheroidal aggregates of DAOY cells, a commonly used human cell line model for medulloblastoma. After just 8 h of 250 V/m dcEF exposure, these aggregates exhibited a significant (p < 0.0001) difference in cathodal bias, yet unlike the U87 mg cells, the movement was toward the anode (−69 ± 12 μm) relative to controls (−9 ± 8 μm) (Fig. 3a). For the DAOY aggregates, the outward movement of the anodal frontier (34 ± 9 μm) was larger, but not statistically different than in the controls (20 ± 9 μm), however the difference in outward growth of the cathodal frontier was significant (−35 ± 10 μm relative to 11 ± 5 μm in controls). This suggests that there may be stronger sensing, or perhaps a repulsive effect, of the dcEF at the trailing end of the DAOY aggregates. The bulk of the cathodal bias in the U87 mg aggregates also came from the change in the cathodal frontier, but with a different polarity of response.

With an extended dcEF exposure for 24 h, the DAOY aggregates, like their 8 h counterparts, exhibit a significant difference relative to controls in outward growth of cathodal frontier (p < 0.0001) but not the anodal frontier, yet maintain their anodal bias (−81 ± 8 μm relative to 0 ± 12 μm in control, p < 0.0001) (Fig. 3b,c). One major difference to the 8 h experiments is that the DAOY aggregates now exhibit significant retraction in the orthogonal frontiers, which results in a significantly smaller spheroidal area after 24 h (p < 0.0001) (Fig. 3d), whereas, for the U87 mg cells, the area of the aggregate bounding box is significantly larger than the control (p = 0.0133), seen as an elongation parallel the dcEF field lines (Fig. 3e).

Given these electrotactic responses, we next investigated possible mechanistic pathways by which U87 or DAOY cells respond to electric fields. Currently, there is still no general consensus on how a dcEF is sensed, how an electrotactic direction is determined, nor how the directional persistence is maintained. In our opinion, the literature on a mechanistic understanding of tumor cell electrotaxis is somewhat under developed, and the application of contemporary transcriptomic methods may serve to improve our understanding.

### Determination of electrotaxis-elicited phenotypes using high-throughput qRT-PCR and RNA sequencing

Directed cell migration and the mechanisms that underlie its processes are complex, multimodal, and often cell-specific—electrotaxis is no different^15,16,35^. The individual mechanisms that have been studied or found to be in some way involved in electrotaxis have been broad and difficult to generalize from: e.g., alternatively-spliced channel expression^20^, inositol-phospholipid signalling^36^, growth-factor receptor electro-polarization^37^, purinergic receptors^38^, lipid rafts^39^, and glycocalyx bending^40^.

While there exist many studies focused on a small number of or single targets, few broad signaling scans on cells undergoing electrotaxis have been performed. In their study of lung cancer electrotaxis, Huang, *et al*., ran microarray analyses finding 431 gene probes (out of 54,675) to be significantly different after dcEF application, with the most significantly represented signaling pathways being those for adherens and tight junctions, and hTerc transcriptional regulation^41^. Li, *et al*., included a 60-protein phosphorylation antibody array in their study, which had directed them to their consequent inquiry into dcEF-mediated effects on the ERK pathway^23^. Barcoded Dictyostelium mutants have also been used as a high-throughput method to piece apart electrotaxis mechanisms, through conservation of genes that correlate with electrotaxis hyperresponsitivity^42^ though this method is not yet feasible in mammalian cells. Two recent studies have included ribonucleic acid sequencing (RNA-SEQ) in their mechanistic analysis of electrotaxis, providing insights into how dcEFs impact actin cytoskeletal, mitogen-activated protein kinase (MAPK), and focal adhesion signaling^43,44^ indicating motility but nothing explicitly unique about electrotaxis. Even with all these previous electrotaxis mechanism studies, there is still no general consensus on how a dcEF is sensed, how an electrotactic direction is determined, nor how the directional persistence is maintained.

Our exploration of electrotaxis mechanisms began with a series of qRT-PCR gene arrays. The first was a custom gene array comprised of 25 genes either previously shown or hypothesized to involved in electrotactic machinery (Supplementary Table S1). Gene expression was compared at 2 h and 8 h, relative to unstimulated cells, with significant, differential expression criteria set to p < 0.05 and absolute fold-change of 2 or greater (Supplementary Fig. S2). For these conditions, however, no genes were found to be significantly differentially expressed, even before multiple hypothesis corrections to the p-values.

Next, deciding to expand our search to canonical mechanisms of cell movement, we analyzed gene expression using a commercial PCR array of 84 known human motility genes (Supplementary Table S2, Supplemental Fig. S3). PLCG1 and RHOA (both involved in Rho signaling) were found to be differentially down-regulated in U87 mg cells after 2 h and EGF was observed to be up-regulated after 8 h. The RHO receptor was the only gene found to be differentially expressed in DAOY cells, up-regulated after 8 h. However, after multiple-hypothesis correction was performed on the initial p-values, none of those genes were found to remain significantly differentially expressed.

Initially, it was surprising to see no canonical motility genes differentially expressed under dcEF, when a dramatic change in motility behavior was clearly observed, particularly when previous work by Yao, *et al*. and Li, *et al*. showed these outcomes^43,44^. However, what is distinct about our specific electrotaxis assay relative to previous work, is that the cells are embedded in a richly nutritional, adhesive matrix, and even without dcEF, the cells are invading directionally—away from the center of the cellular aggregate—yet equally outward in all directions. What this means is that more so than previous electrotaxis assays, where cells have gone from random or non-motile behaviors, our assays must be considered as moving from one form of directed invasion to another. This suggests that the dynamics of electrotaxis-induced gene expression are likely subtler than the previously seen dynamic changes in motility machinery, as our observed gene expression changes will more likely relate to the change of a particular cue, than a complete change in machinery for a different mode of invasion. Our observation is supported by recent work by Bashirzadeh, *et al*., who by using pharmacological modulation of actin and myosin on cells undergoing electrotaxis, showed that electrical fields likely induce changes in polarization and not motility^45^. Based on this evidence, we would expect not to see dramatic changes in motility machinery when the cells are motile prior to dcEF application, which is exactly what we have observed. Thus, we continued our study with a more powerful mRNA analysis tool in order to probe this effect with more subtlety: RNA-SEQ.

**Figure 4:**
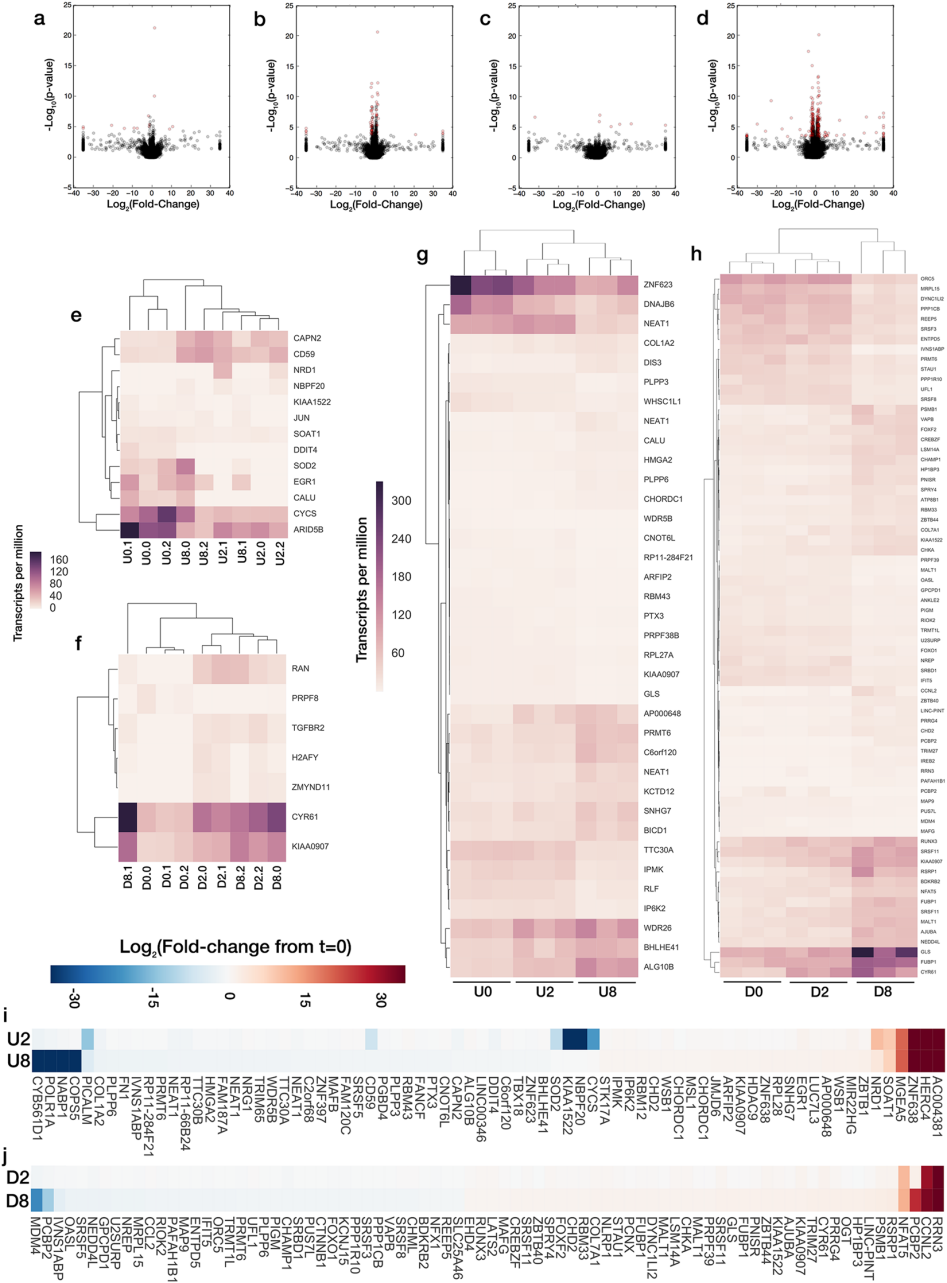
Differential expression of transcripts from RNA-SEQ. (**a**–**d**) Volcano plots comparing fold-change and p-value of each identified transcript accession for (**a**,**b**) U87 mg and (**c**,**d**) DAOY cellular aggregates for samples exposed to 250 V/m dcEFs for (**a**,**c**) 2 h, or (**b**,**d**) 8 h compared 0 h, unexposed controls. Significance threshold is set to FDR < 0.05 and a Log_2_(fold-change) of** > **1 or < −1 (n = 3 for all conditions). Differentially expressed transcripts are shown in red. Clustermaps for transcripts per million of differentially expressed transcripts for: (**e**) U87 mg after 2 h; (**f**) DAOY after 2 h; (**g**) U87 mg after 8 h; (**h**) DAOY after 8 h; relative to 0 h unexposed controls. Each column represents a single replicate sample (U = U87 mg, D = DAOY). Note: for 8 h results, not all differentially expressed transcripts are shown, as Daoy significance cutoff was set to p < 0.0005 and U87 mg to p < 0.005 for improved readability. (**i**,**j**) Fold-change of most significantly differentially expressed transcripts. Comparing (**i**) U87 mg, or (**j**) DAOY differentially expressed genes for 2 h, or 8 h of dcEF relative to 0 h unexposed controls (U = U87 mg, D = DAOY). Not all differentially expressed transcripts are shown, as Daoy significance cutoff was set to p < 0.001 for improved readability.

We performed RNA-SEQ on U87 mg and DAOY spheroidal aggregates 0 h, 2 h, and 8 h after being exposed to 250 V/m dcEFs (n = 3 for each condition). Over 91% of all mRNA transcript reads were successfully assigned to 145,327 unique transcript accessions. Differential expression—comparing either the 2 h or 8 h transcripts against the control, or 0 h transcripts—was based on cutoff criteria of a false-discovery rate (FDR) < 0.05 and an absolute fold-change of 2 or greater (Fig. 4, Supplementary Dataset 1: D1_consolidated_sleuth_output.tds, GEO Accession: GSE115509). Both the 8 h conditions had substantially more differentially expressed transcripts as was to be expected, and the DAOY 8 h comparison yielded 4.5x as many differentially expressed transcripts as the U87 mg 8 h comparison. Over 75% of all differentially expressed transcripts were for protein coding mRNA and the remaining were mostly ‘processed transcripts’ (transcripts without an open reading frame) or ‘retained introns’, mapping to purported splicing variant transcripts.

Using the differentially-expressed transcripts, we performed a pathway over-representation analysis, finding several such over-represented pathways for all conditions except for the U87 mg 2 h set. For the DAOY 2 h condition, only CYR61 and TGFBR2 were found in any pathway annotations, though with so few genes used as the basis for a search, the pathways found to be overrepresented—atrioventricular valve development & morphogenesis, and TGFB-receptor type II homodimer complex—are likely false positives for the current study. Selected pathways from this analysis are listed in (Table 1). Both the U87 8 h and DAOY 8 h conditions yielded significant over-representation for mRNA metabolism, and gene expression regulation. A substantial number of transcription factors were also identified as being significantly overrepresented (Supplementary Table S3). Additionally, the pathways for establishment of RNA localization, cell stress, and mRNA splicing via spliceosome were overrepresented in DAOY 8 h genes. mRNA localization, for ß-actin in particular, has been previous implicated as a factor in directed cell migration^46^. Additionally, ATPase regulator activity was significantly overrepresented in U87 8 h genes, which was previously shown to occur in tandem with ß-actin localization at the leading edge in electrotaxing calvarial osteoblasts^47^. However, further analysis would be required to determine which, if any, mRNAs are being re-localized to this effect.

**Table 1:** Selected significantly over-represented pathways for U87 mg and DAOY undergoing 8 h electrotaxis.

U87 mg 8 h			DAOY 8 h		
term ID	term name	p-value	term ID	term name	p-value
GO:0060590	ATPase regulator activity	0.000281	GO:0044260	Cellular macromolecule metabolic process	1.2E-09
GO:0044260	Cellular macromolecule metabolic process	0.000142	GO:0033554	Cellular response to stress	0.000482
GO:0010467	Gene expression	0.00000718	GO:0051236	Establishment of RNA localization	0.0447
GO:0016071	mRNA metabolic process	0.000457	GO:0010467	Gene expression	1.23E-08
GO:0010468	Regulation of gene expression	0.0000855	GO:0016071	mRNA metabolic process	0.00109
			GO:0000398	mRNA splicing, via spliceosome	0.00042

Genes that were found to be differentially expressed after 8 h for both U87 mg and DAOY were well correlated to each other, with a Pearson’s correlation coefficient of 0.9397 (p < 7.9 × 10^−19^). 5 pathways were found to be significantly over-represented using this gene subset: RNA splicing, gene expression, RNA metabolic process, and two transcription factor pathways, ETV7 and E2F1. This suggests that if there is indeed a conserved transcriptional machinery in response to dcEF exposure, it may lie in regulation of RNA splicing and processing. We also explored transcripts that were differentially expressed exclusively in one cell type, and transcripts that had opposite directions of regulation across the two cell types. These genes were of interest as candidates for describing the difference in directional preference between the cathodally-directed U87 mg and anodally-directed DAOY cells, though no pathways were found that were significantly overrepresented by either subset of these genes.

We next performed a gene-set enrichment analysis using all transcripts with custom ranking from most significantly up-regulated to most significantly down-regulated (i.e., negative log-transformed p-values, signed by direction of their fold-change). Three of the msigDB gene sets were used, CURATED, GENE ONTOLOGY (GO), and ONCOGENIC. Significance criteria was p-value < 0.05 and the GSEA tool recommended false-discovery rate of 0.25.

While there is not sufficient space in this paper to present all of the significantly enriched gene sets (see Supplementary Dataset 2: D2_consolidated_GSEA.xlsx), we have culled the lists for pathways we hypothesize, or that have been previously shown, to be relevant to electrotaxis machinery. We found only a few pathways that matched the 2 previous RNA-SEQ studies for electrotaxis, which could be due to our cells being in 3D or plated as spheroids in invasion-promoting matrix. Li, *et al*. had found the broad cytokine-cytokine receptor and chemokine signaling pathways to be differentially regulated, however, neither of these were found enriched for any of our conditions^44^. KEGG focal adhesion and MAPK signaling pathways were found to be up-regulated, but only in DAOY cells, yet this matching the findings of Yao, *et al*. for Schwann cell electrotaxis toward the anode^43^.

Several signatures of chemotaxis were also present. Most strikingly, all conditions showed up-regulation of genes matching data from Mili, *et al*., a study that characterized RNAs that were localized to cell protrusions under chemotaxis and haptotaxis^48^. RNA localization was also significantly enriched for all dcEF conditions. There were also many pathways enriched relating to the phosphoinositide 3-kinase (PI3K) pathway, a pathway highly involved in intracellular regulation of chemotaxis and previously shown to be a mediator of keratinocyte, neutrophil, and neural progenitor cell electrotaxis^36,49^. The PI3K pathway is of particular interest with respect to the hypothesis that electrotaxis is just another form of chemotaxis—the notion that electrotaxis is just electrophoresis of extracellular molecules that redistribute into a chemotactic signaling gradient. The pathway is also important for the production and maintenance of intracellular signaling gradients, and is part of the signaling that often impacts the specific directionality of a given cell. Related pathways found to be significant via our analysis include the Reactome PI3K cascade in both U87 mg and DAOY cells after dcEF exposure, and gene ontology for phosphatidylinositol (3,4,5)-triphosphate in DAOY cells. Looking at pathways well-connected to PI3K signaling, mechanistic target of rapamycin (mTOR) genes were also enriched, as were pathways for rapamycin sensitive genes, mTOR signaling, and the Insulin-like growth factor 1 (IGF1)/mTOR pathways in both cell lines.

Several growth-factor-associated pathways were also up-regulated for both cells including Reactome pathways for EGFR, fibroblast growth factor receptor, Insulin receptor, transforming growth factor Beta (TGFB), and nerve growth factor, and the pathway interaction database pathways for MET, platelet-derived growth factor Beta, TGFB receptor, and vascular endothelial growth factor 1 and 2. All conditions also displayed positive enrichment for genes that previously had been found to be co-regulated by hepatocyte growth factor (HGF) and Ki-Ras oncogene activity^50^. ErbB and EGFR signaling were also well represented by the significantly enriched gene sets. Both DAOY and U87 mg cells under dcEFs were enriched for the gene ontology for regulation of ErbB signaling as well as the pathway interaction database ErbB1 downstream gene set. Enriched genes for both DAOY and U87 mg cells under dcEFs also reflected data for genes affected by ErbB ligands, EGF & neuregulin 1^51^. The ErbB pathway is of particular interest as it has previously been shown that the electrotactic response in breast cancers appears to correlate epidermal growth factor receptors (EGFR) expression^21,52,53^.

Overall, we found many pathways that were significantly impacted by the dcEF, ranging from chemotaxis, to growth factor signaling, and to intermediate signaling activity of the PI3K and mTOR pathways. Yet, just because a pathway is over-represented or enriched does not guarantee it will be causally linked to electrotaxis. Thus, we next performed functional validation based on some of these hypothetical mechanisms using pharmacological inhibition.

### Evaluating putative electrotaxis signaling pathways using pharmacological inhibition

**Figure 5:**
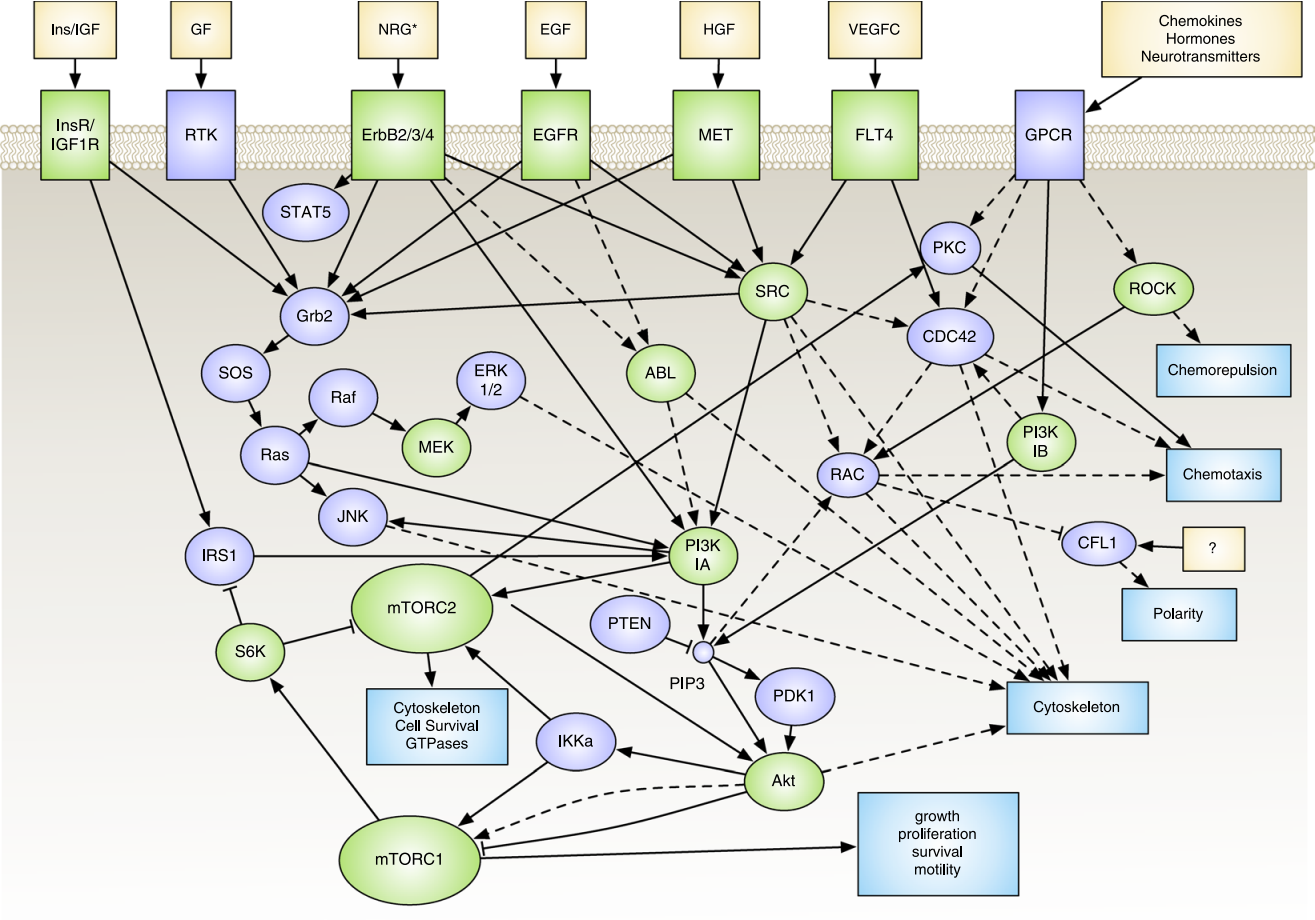
Abridged pathway network for selected targets. Inhibited components are highlighted in green. Data obtained from multiple KEGG pathways^72^.

Our study of electrotaxis mechanisms continued with the use of pharmacological agents to inhibit specific likely pathways involved in electrotaxis to identify critical pathways involved. Specifically, we explored the role of the PI3K pathway as a nexus (Fig. 5), since many of our transcriptomic findings, as well as previous electrotaxis studies, have pointed to this pathway as an intermediate regulator for transducing the sensing of a dcEF into the persistent and directional migration of a cell.

We characterized the invasive responses of DAOY and U87 mg cellular aggregates under exposure to various pharmacological inhibitors with and without dcEF. The inhibitors we have chosen and their corresponding targets are listed in Table 2. In these experiments, either DAOY or U87 mg spheroidal aggregates were exposed to an inhibitor for an initial 24 h, after which the extent of electrotaxis was measured for 24 h.

**Table 2:** Pharmacological Inhibitors used to explore electrotaxis pathways. Note, some compounds were used at multiple doses (highlighted in **bold**).

Drug	CAS#	Target(s)	Dose(s) (uM)
Isoginkgetin	548-19-6	pre-mRNA splicing	33
AZD8931	848942-61-0	EGFR, ErbB2/3	**1, 10**
OSI-906	867160-71-2	IGF1R, InsR	1
Bosutinib	380843-75-4	Src, Abl	1
KU-0063794	938440-64-3	mTORC1/2	2.5
MK-2206	1032350-13-2	Akt1/2/3	2
Foretinib	849217-64-7	Met, KDR, Tie-2, VEGFR3/FLT4	0.5
PD0325901	391210-10-9	MEK	0.5
LY294002	154447-36-6	PI3Kα/δ/β	**2, 20**
BEZ235	915019-65-7	PI3Kα/δ/β/γ, mTOR, ATR	**0.025, 0.25**
Rapamycin	53123-88-9	mTOR	0.1
Erlotinib	183319-69-9	EGFR	**5, 50**
CZC24832	1159824-67-5	PI3Kγ	1
Mubritinib	366017-09-6	ErbB2	1
Y-27632	129830-38-2	ROCK1/2	10
AZ5104	1421373-98-9	EGFR, ErbB4	0.006

Supplementary Fig. S4 displays the proportional change in spheroidal bounding box for the controls without dcEF, from 24 h to 48 h after exposure to each inhibitor. Only rapamycin’s effect on the U87 mg cells was found to be significantly different than controls (p = 0.0039) with a negative proportional change in area over that time period. Additionally, viability data is also provided in Supplementary Fig. S5 for our initially tested inhibitors. The mTOR1/2 inhibitor, KU-0063794, significantly affected viability over 24 h exposure for both U87 mg and DAOY cells, though it was at a level on the order of the amount of growth for no additional proliferation, and morphologically, the cells did not appear to be distressed. The pan-AKT inhibitor, MK-2206, had a similar outcome for the U87 mg cells. It is important to note these findings as they may constrain our claims when it comes to the electrotactic effects on the cells with those inhibitors.

**Figure 6:**
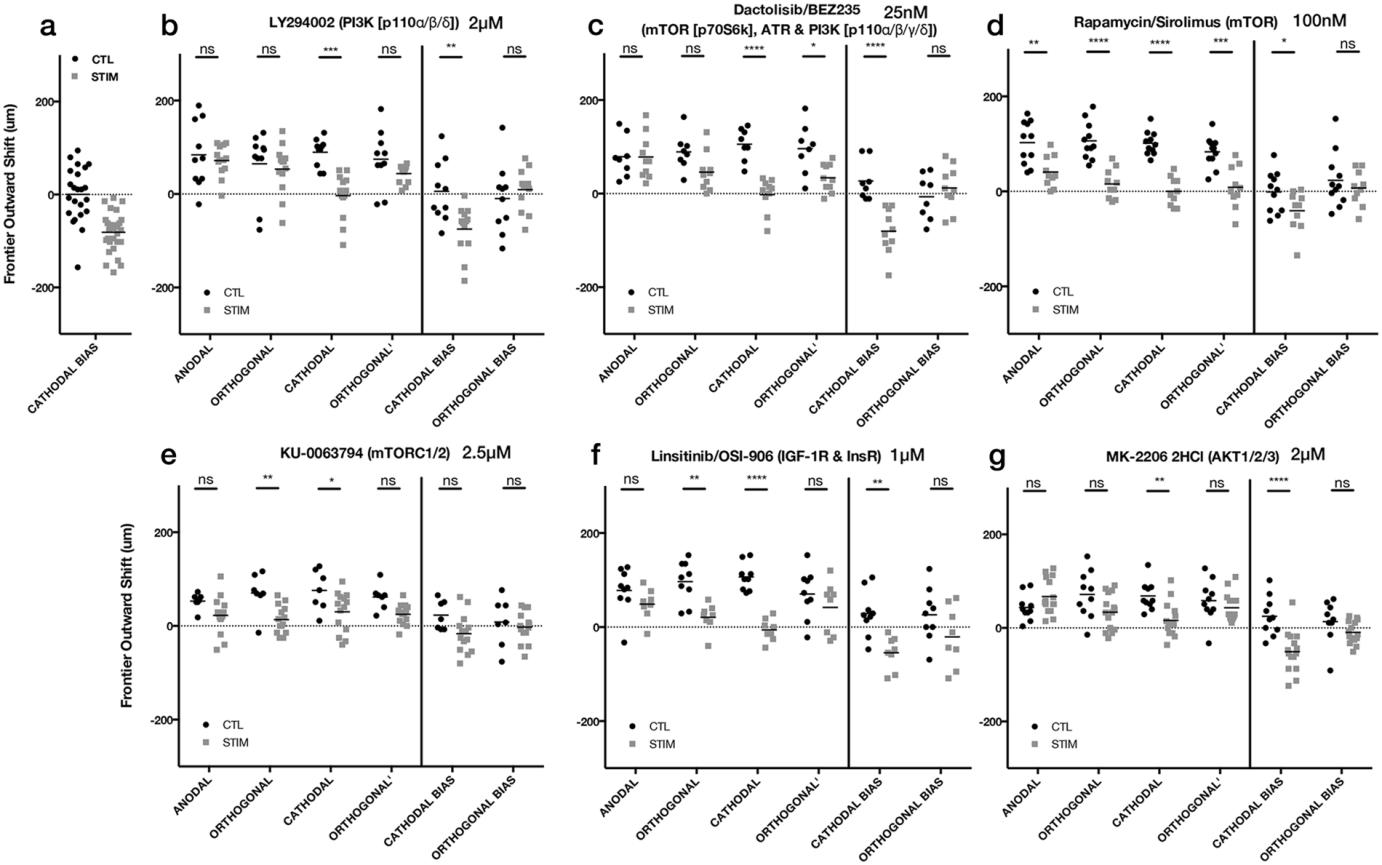
Effect of PI3K/mTOR/IGF/AKT inhibitors on DAOY spheroidal aggregates undergoing electrotaxis. 24 h hours after dcEF (stim) or controls without dcEF (CTL) (**a**) 24 h DAOY data without inhibitor compounds, redisplayed from Fig. 3b. (**b**) LY294002 (2 μM) ***p = 0.0003, **p = 0.0020 (CTL, n = 10; STIM, n = 13); (**c**) BEZ235 (25 nM) *p = 0.0112, ****p < 0.0001 (CTL, n = 8; STIM, n = 10); (**d**) Rapamycin (100 nM) **p = 0.0014, *p = 0.00465, ****p < 0.0001 (CTL, n = 11; STIM, n = 10); (**e**) KU-0063794 (2.5 μM) **p = 0.0057, *p = 0.0351 (CTL, n = 7; STIM, n = 14); (**f**) OSI-906 (1 μM) **p = 0.0034 (orthogonal), **p = 0.0013 (cathodal bias), ****p < 0.0001 (CTL, n = 9; STIM, n = 8); (**g**) MK-2206 (2 μM) **p = 0.0040, ****p < 0.0001 (CTL, n = 10; STIM, n = 15); ns = not significant; Two-way ANOVA, Holm-Sidak post-hoc.

We began with direct inhibition of the PI3K pathway. For DAOY cells, neither the pan-PI3K inhibitor, LY294002 (2 μM) nor BEZ235 (25 nM), appeared to affect the electrotactic bias (Fig. 6b,c). Although direct inhibition of PI3K was not impacted for DAOY, we continued our study by moving downstream to the mTOR pathway, which is also involved in many aspects of cell growth and invasions, sometimes independently of PI3K signaling. The mTOR inhibitor, rapamycin (100 nM), appeared to increase overall outward growth of the DAOY cells, however the anodal bias was still preserved (Fig. 6d). Inhibition of mTORC1/2 with KU-0063794 no longer exhibited an anodal bias, however this was one of the conditions where viability was affected, and there appears to be an overall decrease in outward frontier growth (Fig. 6e). We also tested DAOY cells against canonical PI3K upstream and downstream targets, but neither IGF inhibition with OSI-906 (1 μM), nor pan-AKT inhibition with MK-2206 (2 μM), had an effect on these cells (Fig. 6f,g).

**Figure 7:**
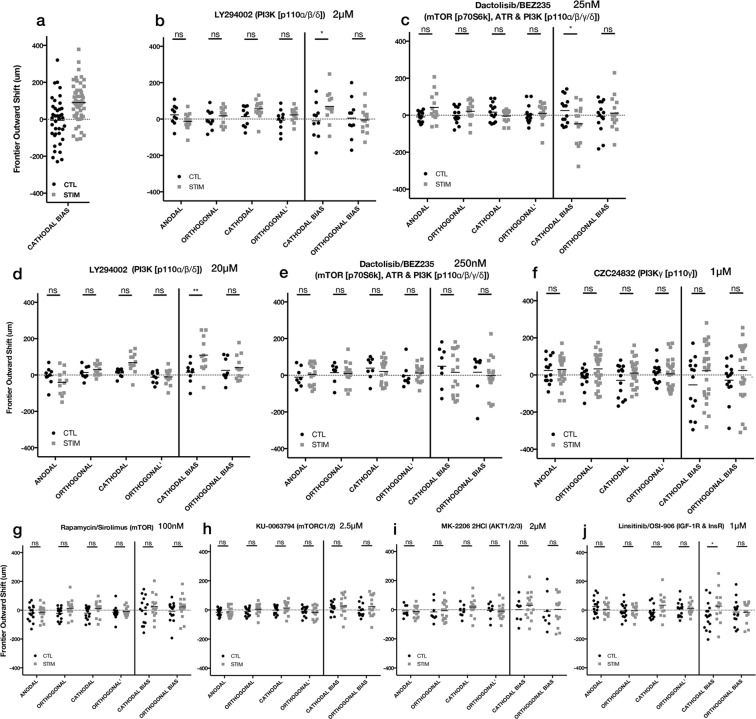
Effect of PI3K/mTOR/IGF/AKT inhibitors on U87 mg spheroidal aggregates undergoing electrotaxis. 24 h hours after dcEF (stim) or controls without dcEF (CTL) (**a**) 24 h U87 mg data without inhibitor compounds, redisplayed from Fig. 2d; (**b**) LY294002 (2 μM) *p = 0.0269 (CTL, n = 10; STIM, n = 13); (**c**) BEZ235 (25 nM) *p = 0.0306 (CTL, n = 14; STIM, n = 14); (**d**) LY294002 (20 μM) **p = 0.0015 (CTL, n = 9; STIM, n = 12); (**e**) BEZ235 (250 nM). (CTL, n = 8; STIM, n = 17); (**f**) CZC24832 (1 μM). (CTL, n = 14; STIM, n = 26); (**g**) Rapamycin (100 nM) (CTL, n = 15; STIM, n = 15); (**h**) KU-0063794 (2.5 μM) (CTL, n = 13; STIM, n = 15); (**i**) MK-2206 (2 μM) (CTL, n = 8; STIM, n = 16); (**j**) OSI-906 (1 μM) *p = 0.0479 (CTL, n = 16; STIM, n = 13); ns = not significant; Two-way ANOVA, Holm-Sidak post-hoc.

Unlike the DAOY cells, however, the U87 mg cells required aspects of the PI3K signaling pathway for electrotaxis. Pan-PI3K inhibition resulted in a loss of significant difference in the outward movement of the cathodal frontier, and, although it was still significantly different, the cathodal bias under LY294002 (2 μM) inhibition appeared slightly diminished relative to no inhibitor controls (Fig. 7b). Surprisingly, BEZ235 (25 nM) inhibition led to a small significant difference in cathodal bias, but the directionality was now slightly anodal (Fig. 7c).

A follow-up study was run with the same inhibitors (LY294002 and BEZ235), but this time with 10x higher dose (Fig. 7d,e). The results for LY294002 (20 μM) were consistent with the smaller dose, and seemed to have no overall effect on the cathodal bias. For BEZ235 (250 nM), the bias was no longer anodal, however, any electrotactic bias was gone, and the frontiers appeared to be no different than the unstimulated controls.

There are only a few targets that don’t overlap between LY294402 and BEZ235, one of those being PI3Kγ, unique from other PI3Ks in that it is an intermediate in the G-protein coupled receptor pathway (GPCR)—whereas PI3Ks α, β, and δ are more strongly tied to receptor tyrosine kinase signaling. We used a PI3Kγ-specific inhibitor, CZC24832 (1 μM), to test the hypothesis that this was the particular target that was necessary for producing an electrotactic bias (Fig. 7f), and indeed, specific inhibition of PI3Kγ appeared to also eliminate any electrotactic bias. This strongly suggests that PI3Kγ signaling and potentially upstream signaling from GPCRs play a role in the electrotaxis of U87 mg cells. This also expands upon previous work by Zhao, *et al*., and Meng, *et al*., where knock-out of PI3Kγ in murine keratinocytes, neutrophils and neural progenitor cells all showed attenuated cathodal responses to dcEFs in wound healing assays^36,49^.

We continued our exploration of the role of PI3K signaling in U87 mg electrotaxis finding that inhibition of mTOR and mTORC1/2 both led to abrogation of any electrotactic bias (Fig. 7g,h) as well as pan-AKT inhibition (Fig. 7i), all of which are downstream effectors of PI3K. Upstream of PI3K, IGF inhibition did not impact cathodal bias (Fig. 7j), which was expected as IGF signaling requires the PI3K pathway that includes PI3Ks α, β, and δ.

Another pathway for which we observed broad regulatory changes in our RNA-SEQ data was the ErbB pathway. This family of surface receptors is known to form hetero- and homo-dimers on the cell surface as sensors for various ligands. EGFR has been previously studied for its role in electrotaxis, as mentioned above, yet the other ErbB isoforms have not. We began our study by targeting 3 of the 4 main ErbB2 isoforms, EGFR (or ErbB1), ErbB2 (or HER2), and ErbB3 (or HER3).

Inhibition of ErbB signaling with either Erlotinib (5 μM) nor AZD8931 (1 μM) seemed to not affect the electrotactic bias of DAOY cells (Supplementary Fig. S6). However, the outward growth of each frontier was greatly diminished relative to non-inhibitor controls. Yet with the electrotactic bias still in place, this could merely be due to a diminished source of general growth signal from the ErbB receptors.

**Figure 8:**
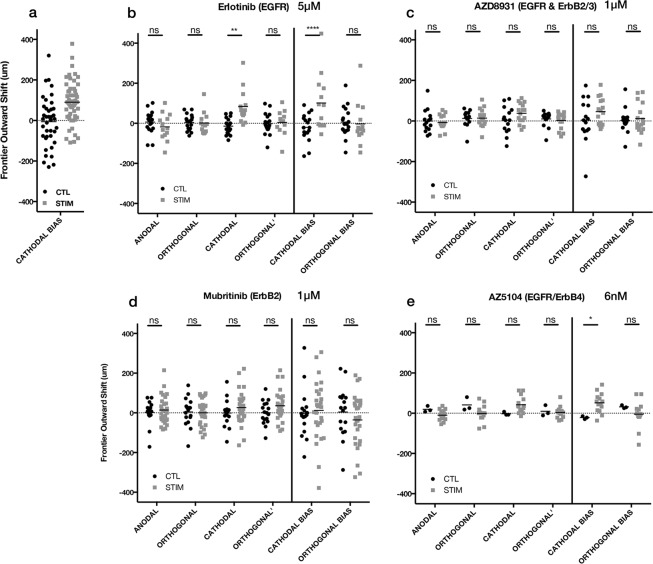
Effect of ErbB inhibitors on U87 mg spheroidal aggregates undergoing electrotaxis. 24 h hours after dcEF (stim) or controls without dcEF (CTL). (**a**) U87 mg data without inhibitor compounds, redisplayed from Fig. 2d. (**b**) Erlotinib (5 μM) **p = 0.0016, ****p < 0.0001 (CTL, n = 18; STIM, n = 13); (**c**) AZD6931 (1 μM). (CTL, n = 15; STIM, n = 16); (**d**) Mubritinib (1 μM).(CTL, n = 17; STIM, n = 29); (**e**) AZ5104 (6 nM) *p = 0.0386 (CTL, n = 3; STIM, n = 14); ns = not significant; Two-way ANOVA, Holm-Sidak post-hoc.

The U87 mg cells appeared to be unaffected by EGFR inhibition (Fig. 8b), however, the cellular aggregates grew no differently than controls with the pan-ErbB inhibitor AZD8931 (1 μM), and had lost their cathodal bias entirely (Fig. 8c). Testing both ErbB inhibitors at 10x increased doses, yielded the same effect (Supplementary Fig. S7).

We were unable to procure a pharmacological inhibitor for ErbB3-specific inhibition, but we did attempt to determine whether ErbB2-specific inhibition with Mubritinib resulted in loss of electrotactic bias, and surprisingly, it did (Fig. 8d). This is notable, because U87 mg cells are generally considered ErbB2-negative or ErbB2-weakly-expressing^54^. We hypothesized that the U87 mg cells, as some tumor cells have been shown to do previously^55^, up-regulated their ErbB2 expression when they were plated in 3D, dispersed or as aggregates, which would also explain the tempered electrotactic response we observed for 2D-dispersed scenarios. This was not the case however, as flow cytometry of the U87 mg in 2D vs. 3D, dispersed vs. aggregated, yielded no change in ErbB2 expression relative to an isotype negative control, and did not approach the signal of ErbB2+ MCF7 breast cancer cells (Supplementary Fig. S8). Simultaneous inhibition of EGFR and ErbB4 did not impact U87 mg electrotactic bias (Fig. 8e). As per our study, it is only clear that intact ErbB2 signaling is required for U87 mg electrotaxis, though whether this is through ErbB2 homodimers or ErbB2-ErbB3 heterodimer signaling, or some other undiscovered form, and whether this is ligand-, charge- or physiomechanically-mediated is still unknown.

Lastly, we tested a few additional inhibitors for various targets that were found to be differentially-regulated after dcEF in the RNA-SEQ data. Both the Src and HGF pathways are of interest in that their activity would support a chemotactic-overlap hypothesis for electrotaxis. The Src/Abl pathway acts upstream of PI3K and receives input from a number of receptor molecules including EGFR, signals of oxidative stress, and growth factors^56^. Our RNA-SEQ analyses produced several significantly enriched gene sets for ERK signaling, which has also been previously implicated in electrotaxis^57^, thus we have also chosen, PD0325901, a MEK inhibitor that acts upstream of ERK signaling. We also saw numerous gene sets across both cell lines suggesting that HGF and VEGF signaling were differentially regulated under dcEFs, thus we have included an ATP-competitive inhibitor of both HGFR and VEGFR (mostly for MET, KDR, TIE2, and FLT4). Additionally, we observed a myriad of evidence indicating dcEF-induced RNA localization and splicing. While inhibition or control of RNA localization is beyond the scope of simple pharmacological inhibition, we were able to perform a cursory test on general inhibition of RNA splicing with Isoginkgetin^58^.

**Figure 9:**
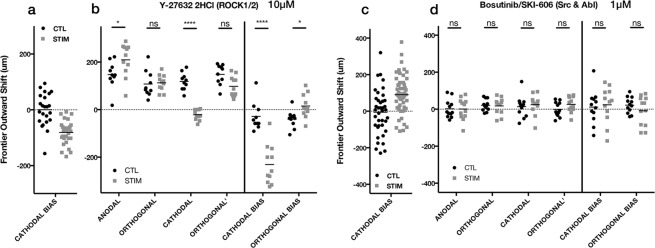
Additional inhibitors that impacted electrotaxis of DAOY (**a**,**b**) or U87 mg (**c**,**d**) spheroidal aggregates. 24 h hours after dcEF (stim) or controls without dcEF (CTL) (**a**) DAOY data without inhibitor compounds, redisplayed from Fig. 3b; (**b**) DAOY + Y-27632 (10 μM) *p = 0.0279 (anodal), *p = 0.0472 (orthogonal bias), ****p < 0.0001 (CTL, n = 10; STIM, n = 11); (**c**) U87 mg data without inhibitor compounds, redisplayed from Fig. 2d; (**d**) U87 mg + Bosutinib (1 μM) (CTL, n = 12; STIM, n = 12); ns = not significant.; Two-way ANOVA, Holm-Sidak post-hoc.

In DAOY cells, both inhibition of MEK with PD0325901 (500 nM) and Src/Abl with Bosutinib (1 μM) led to a loss of significant different in anodal bias relative to controls, however, this may be a technical/statistical artifact, because all but two of the replicates for each had an anodal bias (Supplementary Fig. S9a–d). Inhibition of the pre-spliceosome with Isoginkgetin (33 μM) had no effect on DAOY electrotactic bias, nor did inhibition of HGF/VEGF signaling with Foretinib (500 nM). Since no chemotactic signaling pathway we inhibited was observed to have an effect on DAOY electrotactic bias, we wondered if the mechanism was instead driven by chemorepulsion on the cathodal side. However, this hypothesis was quickly nullified when inhibition of ROCK1/2—regulators of trailing end retraction during migration^59^—using Y-27632 (10 μM) led to a 2.8-fold increase in anodal bias (Fig. 9b). This is surprising, as previously Yao, *et al*. used ROCK inhibition as a means to decrease the speed of hippocampal neurons guided by dcEF—though these neurons were cathodally-directed, and also responded to PI3K inhibition, which the DAOY aggregates did not^60^.

With the four additional inhibitors we tested on U87 mg aggregates (Supplementary Fig. S8e–g), only Src/Abl inhibition with Bosutinib (1 μM) had any effect on the overall cathodal bias (Fig. 9d). Though, this is particularly interesting with respect to our findings from ErbB inhibition, as Src is known to interact with ErbB2 and ErbB3 complexes^61^. However, Src and Abl are, like mTOR and AKT, broadly connected to signaling pathways for many cellular functions, and whether Src, ErbB2/3, mTOR, AKT, and PI3Kγ actually form a mechanistic network in U87 mg electrotaxis requires further confirmation.

**Figure 10:**
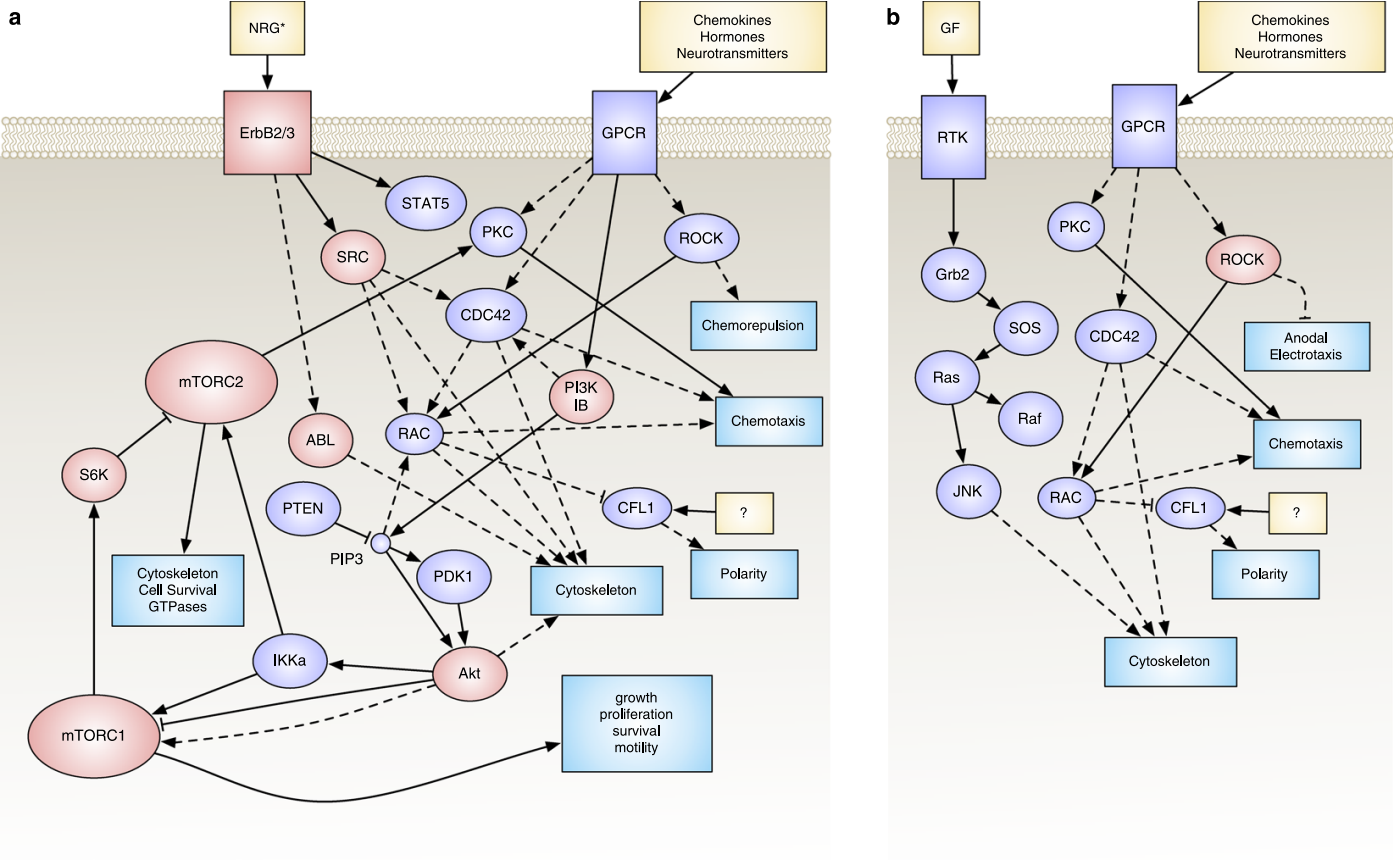
Revised pathway networks after findings from pharmacological inhbition studies. For (**a**) U87 mg; and (**b**) DAOY spheroidal aggregates. Inhibited components that impacted electrotactic responses are highlighted in red.

Overall, while DAOY cells did not appear to be affected by a loss of PI3K, mTOR, IGF, or AKT signaling, the U87 mg cellular aggregates lost their cathodal bias when PI3Kγ was inhibited, yet other PI3Ks (α, β, and δ) do not appear to be regulating electrotaxis (Fig. 10). mTOR and AKT also appear to be part of the electrotactic response in U87 mg cells, though if and how they are connected to PI3Kγ requires further study, as well as determining what input to PI3Kγ is driving the response and why it induces a cathodal response in these particular cells. mTOR and AKT are known to be connected signaling pathways, but both also have broad signaling connectivity across the spectrum of cellular function, thus confirming how the electrotactic signal propagates through the PI3K/AKT/mTOR network also demands further study.

The ErbB pathway also appears to be required for electrotaxis in U87 mg cells, as inhibition of ErbB2 and potentially ErbB3 was able to attenuate the cathodal bias similar to controls without dcEFs. This was surprising as ErbB2 expression in U87 mg cells is known to be low, and we did not see a change in this expression across various plating formats. Which particular hetero- and/or homo-dimers of ErbB2 and ErbB3 are specifically involved requires further study, as well as whether these receptors are undergoing electrophoresis and asymmetrically expressed, as was EGFR in previous electrotactic studies^53^.

None of our four inhibitors for Src/Abl, MEK, pre-spliceosome, or HGF/VEGF signaling seemed to have much effect on DAOY cells. However, the inhibition of ROCK1/2 did dramatically increase the DAOY anodal bias, suggesting that future studies should examine the balancing of various GTPases as a way to attenuate or enhance electrotaxis in these cells. For the U87 mg aggregates, only the Src/Abl inhibitor was able to remove the cathodal bias. This interesting in that, although Src is broadly connected to many cellular functions, it is known to be connected to the other targets we verified to be in part essential for U87 mg electrotaxis.

Given these distinctly different responses for the DAOY and U87 cells, it is yet unclear whether these results are generalizable across more GBM or MB tumors, or if they represent hallmark phenotypes that will generalize across all cells that respond either anodally or cathodally to a dcEF. As we have been able to use RNA-seq to select molecular targets for study, we hypothesize that the mRNA phenotype will be one way to further dissect whether a cell will exhibit electrotaxis, and if so, which direction it will move. While we have seen that our two chosen cell lines move in opposite directions, resulting in different mRNA phenotypes, and thus react differentially to pharmacological intervention, substantial work is still required to further generalize the results in this report.

## Conclusion

Here, we report a platform for 3D electrotaxis that affords flexible plating configuration, simple fabrication, capacity for long-term (>4 h) experiments, and the ability to analyze population-level changes without live-cell microscopy. Using this system, we showed that U87 mg and DAOY cells have opposing electrotactic responses–cathodal and anodal, respectively–when plated as 3D spheroidal aggregates, and both cell types maintain this response for at least 24 h. Null results from qRT-PCR arrays, suggest that electrotaxis as spheroidal aggregates is a redirection of existing invasion signals, effecting polarization and not motility. Bulk RNA-SEQ was performed to further explore underlying mechanisms, revealing several potential pathways of interest including: RNA localization, splicing, ATPase regulation, IGF/PI3K/mTOR/AKT signaling, myriad of growth factor signaling pathways. Functional studies with pharmacological inhibition showed that (1) for DAOY electrotaxis, other than ROCK1/2 inhibition (which elicited an increase in anodal bias), no selected inhibitors were found to alter the anodal bias; (2) the cathodal bias of U87 mg aggregates under dcEF was lost under Src/ABL, AKT1/2/3, mTOR and mTORC1/2 inhibition; (3) ErbB2 (and possibly ErbB3), but not EGFR or ErbB4 inhibition removed the cathodal bias of U87 mg aggregates; and (4) PI3Kγ, but not PI3K α/β/δ inhibition led to a loss of U87 cathodal bias.

Altogether, these studies establish a baseline to investigate the ability of dcEFs to ‘cluster’ or ‘move’ dispersed tumor aggregates in tumor models intentionally as a way of coalescing dispersed brain tumor aggregates and provide potential molecular targets that may be available due to a physiological response to dcEFs.

## Methods

### *In vitro* cell culture and cell lines

U87 mg (human glioblastoma, ATCC HTB-14), DAOY (human medulloblastoma, ATCC HTB-186), and MatLyLu (rat prostate cancer, ATCC JHU-5) cells were obtained from ATCC. MCF-7 (human adenocarcinoma, ATCC HTB-22) cells were obtained from the Duke University Cell Culture Facility, all originally sourced from ATCC. All cells were maintained at 37 °C with 5% CO_2_ in Dulbecco’s Modified Eagle Medium (cellgro 15-017CV) with 10% fetal bovine serum (Gemini 900-108), 1% L-Glutamine (Lonza 17–605 F), 1% Non-essential amino acids (cellgro 25–025-CI), and 1% penicillin-streptomycin (Corning 30-001-CI). Cells were passaged before confluence using 0.25% Trypsin-EDTA (gibco 25200-056). As noted for some experiments, U87 mg and MatLyLu cells were made to stably express enhanced green fluorescent protein (GFP), via transfection with a GFP-expression plasmid using the Effectene Transfection Reagent (Qiagen 301425) and further selection of stable transfectants with G418 Sulfate (Gemini 400-113).

### Construction of electrotaxis chambers

Corning 6-well plates (3516) were used as the basis for the chambers. The center two wells served as the plating region and media reservoirs. The outer four wells served as buffer reservoirs and electrode-electrolyte transition wells. The three wells of each row were coupled so that two assays could be performed on a single plate. Platinum electrodes (Omega Engineering SPPL-008) were used in an electrolyte bath of phosphate buffered solution (Corning 21-040-CV). Electrodes were held in place by stainless steel alligator clips, fastened into the well-plate lid with silicone sealant (Dow Corning 734) and used for external power-supply connection.

Direct application of sustained DC fields can elicit harmful effects on cells and tissues through the production of cytotoxic elements at the electrode-electrolyte interface^62^. While platinum is considered relatively stable for these applications, chambers were connected via agar-salt bridges in order to further filter out potentially harmful electrode products. These bridges were comprised of 1/8″ silicone tubing (McMaster-Carr 51135K11), filled with 5% autoclaved agar (BD Difco 214530), 1 M KCl (VWR BDH0258), and 25 mM HEPES (Sigma, H4034). Polydimethylsiloxane (PDMS) “clips” were used to maintain tube curvature. Pre-fabricated tubes were stored in autoclaved deionized water with 100 mM KCl until use. 1/8″ holes were drilled into the lid of the well-plate such that the bridges could be inserted to connect each electrolyte well to the nearest media reservoir.

The plating area was a channel constructed such that the height of the plating area was 202 μm high, 5 mm wide and 19 mm long. Walls of the plating area were built from two layers of double-sided solvent-resistant tape (polyethylene, McMaster-Carr 7602A56), cut to fit the curvature of the well-plate and centered so that there was sufficient space for media reservoir on either end of the plating area channel. The plating area was topped by PDMS (Sylgard 184) strips, made to match the curvature of the well-plate and cut to fit the width of the tape walls. These were made sufficiently high and sealed to the side of the well with autoclaved vacuum grease (Dow Corning, high-vacuum silicone grease) such that the electrical current could only travel through the plating area channel when applied. COMSOL Multiphysics 4.4 was used to simulate current density in the channel using electrolyte conductivity of 1.5 S/m.

### Preparation of spheroidal cell aggregates

A microwell-based method was used for producing spheroidal aggregates. PDMS reverse molds were made from Aggrewell 400Ex (StemCell Technologies 27840). These were used to cast 3% autoclaved Agar (BD Difco 214530) versions of the original Aggrewells. The agar versions were placed in 6-well plates and sterilized under ultra-violet light before use. Cells were counted and diluted to either 700 K or 1.4 M cells per 3 mL—unless otherwise specified—and transferred in 3 mL volume to each well. Plates were centrifuged for 4 min at 280 × g and then transferred to 37 °C, 5% CO_2_ and left for 24 hours to grow before experimental use.

Aggregates were aspirated from the plates, both concentrations combined, diluted to concentrations where individual spheroids could be discriminated under microscope in a single 2D plane, and added to electraxis channels with a 1:1 ratio of aggregates/media to Growth-Factor-Reduced Matrigel (Corning #354230) in 30 μL total volume (note: Matrigel batches were kept consistent within each set of experiments). The solution was allowed to solidify for 30 minutes before media reservoirs were filled. Cells were given 24 h to acclimate to 3D conditions before application of electrical fields.

### Application of electrical fields

Electrical fields were applied to each of the alligator clips on the electrotaxis chambers via a simple series circuit of 27 V battery supply, and a 1MΩ potentiometer. Current through the system was measured with a digital multi-meter, as the potentiometer was adjusted in order to provide the desired field strength as calculated by Ohm’s law in this form:

$${I}_{set}=E\ast \sigma \ast A$$

Where: *E* is the desired field strength (in *V/m*); *σ* is the conductivity of the media (in *S/m*; set to 1.5 S/m for all included studies); *A* is the cross-sectional area of the electrotaxis channel, orthogonal to the flow of current (in *m*); and *I*_*set*_ is the set current measured by the multimeter (in *A*).

During ongoing stimulation, periodic checks are made: (i) to ensure set currents have remained consistent (note: in this study, we kept currents within ranges that would indicate a ±20 V/m per 4 h drift in field strength)—experiments that do not meet this criteria are excluded from further analysis; (ii) to reset I_set_ to desired value; (iii) to replace media if any visually-detectable (by phenol red) pH shift gradient has formed; and (iv) to replace salt-bridges. The determination of this check period duration is done by small pilot studies for each experiment and depends mainly on the amount of current applied.

### Microscopy

Images were taken with either a Zeiss Axiovert 200 using Microlucida (v2.50.2a, MBF Bioscience) or a Leica DMi8 with Leica Application Suite X tile scan. Plates were all aligned in the microscope such that the anodal side (direction toward positive battery terminus) is to the left of the image. Z plane focus was set to the middle depth of the channel.

### Single cell analysis

Single cell experiments were imaged under temperature control for up to 4 h. Images were analysis using the manual-tracking tool in ImageJ (FIJI). Displacement toward the cathode was calculated by taking the scalar projection (|A| cos θ) of each segment length (|A|) where θ is the angle between cell’s path vector (A) and the electrical field line aligned toward the cathode (y = 0 for x > 0). Displacement magnitudes are then normalized by the duration between the initial and final time points for each segment and reported as an average of all segments for a given cell.

### Spheroidal analysis

Tiled images of plating channels containing spheroidal aggregates were taking before and during each experiment. Without altering scale or image content, tiled images were re-aligned across multiple time points in Adobe Photoshop using either registration marks made with solvent-resistant markers on the underside of the well, or by unique patterns in the tape edges that line the stimulation channel. Control wells received no dcEF. Individual aggregates were selected from the tiled images and cropped for further analysis. A conservative frontier-based method was used for determining the extent of each aggregate and demarcated for each of four directions relative to the alignment of the dcEF. The absolute outward shift of each frontier over time is reported for each aggregate. Cathodal bias is defined as the outward shift of the cathodal frontier minus the outward shift of the anodal frontier. Similarly, the orthogonal bias is the outward shift of the orthogonal minus the orthogonal’ frontiers.

The bounding box formed from the frontiers is also used for reporting size of the aggregates as they change over time; these are normalized to the size of the initial frontier bounding box at time = 0 and reported as a proportional change in area.

### qRT-PCR: cell culture and preparation

U87 mg and DAOY spheroidal aggregates were formed using 700 K cells in 3 mL per agar aggrewell. Aggrewells were incubated for 24 h and then spheroids were transferred to electrotaxis chamber and embedded in 1:1 cell culture medium to reduced-growth-factor Matrigel (Corning 354230) and allow to acclimate for 24 h before stimulation. Cells were stimulated at 250 V/m for 0, 2, or 8 h. Electrotaxis channels were scraped and the cells and Matrigel collected into 600 μL of cell recovery solution (BD, now Corning 354253) and kept on ice for 30 min. To provide sufficient RNA for analysis, 2 technical replicates were run in tandem for each biological replicate, and then pooled together, for a total of 4 biological replicates for each condition (except 8 h DAOY which only had n = 3, due to poor RNA quality in one sample). Pools were centrifuged for 5 min at 800xg and resuspended in 75 μL 1% ß-mercaptoethanol (Sigma M3148)/Buffer RLT (Qiagen 79216), vortexed for 1 min, and stored at −80 °C.

### qRT-PCR: RNA isolation and quality control

RNA was isolated using a RNeasy Micro Kit (Qiagen 74004). Sample purity and concentration was assessed on a Nanodrop 2000 (Thermo-Fischer).

### qRT-PCR: primers

Electrotaxis candidate gene assay primers (100 uM) were obtained from Fluidigm. Primers and sequences are listed in Supplementary Table S1. For this assay, ACTB, GAPDH, IPO8, RPL13A, SDHA, and TBP were used as housekeeping genes^63^. For the migration gene assay, a Fluidigm-format Human Motility Array (SABiosciences PAHS-128ZH-1, 20uM) was used. Sabioscience primer sequences are proprietary, however, the genes tested are listed in Supplemental Table 2. For this assay, ACTB, B2M, GAPDH, HPRT1, and RPL13A were provided on the array as housekeeping genes.

### qRT-PCR: cDNA preparation and Fluidigm qRT-PCR

Complementary deoxyribonulcleic acid was produced slightly differently for each assay. For the candidate gene assay a RT2 First Strand kit (Qiagen 330401) was used; for the motility gene assay the RT2 Microfluidics Reagent System (Qiagen 330431) and RT2 PreAMP Pathway Primer Mix for Fluidigm (Qiagen 330241) were used. Fluidigm qRT-PCR was performed on a Fluidigm BioMark Genetic Analysis Platform at the Georgia Tech Genomics Core.

### qRT-PCR: data analysis

qRT-PCR data were analyzed using the SABiosciences RT2 Profiler PCR Array Data Analysis web tool (version 3.5), with default settings other than a geometric mean used for housekeeping gene normalization and a cycle threshold of 35. Data were post-processed and visualized using custom Python (v2.7.x, Python Software Foundation, Anaconda Distribution) code. Multiple hypothesis correction was done with the statsmodel library using the Benjamin-Hochberg method.

### RNA-SEQ: cell culture and preparation

U87 mg and DAOY spheroidal aggregates were formed using 700 K cells in 3 mL per agar aggrewell. Aggrewells were incubated for 24 h and then spheroids were transferred to electrotaxis chamber and embedded in 1:1 cell culture medium to Growth Factor Reduced Matrigel (Corning 354230) and allow to acclimate for 24 h before stimulation. Cells were stimulated at 250 V/m for 0, 2, or 8 h. Electrotaxis channels were scraped and the cells and Matrigel collected into 600 μL of cell recovery solution (BD, now Corning 354253) and kept on ice for 30 min. To provide sufficient RNA for analysis, 3 technical replicates were run in tandem for each biological replicate, and then pooled together, for a total of 4 biological replicates for each condition (except 8 h DAOY which only had n = 3, due to poor RNA quality in one sample). Pools were centrifuged for 5 min at 800xg, supernatant removed and stored at −80 °C.

### RNA-SEQ: RNA isolation, quality control and library preparation

RNA was extracted using a RNeasy Micro Plus Kit (Qiagen 74034). Purity and yield were assessed on a Nanodrop 2000 (Thermo-Fisher). RNA integrity was assessed with an Agilent Bioanalyzer RNA 6000 Nanokit on an Agilent Bioanalyzer 2100. The 3 biological replicates with the best quality control results were chosen for each of the 6 conditions. Library preparation was done with a TruSeq Stranded mRNA Sample Preparation Guide LT with polyA tail pull-down. Library was validated with an Agilent DNA 1000 kit after fragmentation and addition of adapters. Samples were cleaned with AMPure XP beads and normalized with fluorimetric quantification to 10 nM using a Qubit 2.0 Fluorometer using Qubit Broad Range (ThermoFisher Q32850) and High Sensitivity (ThermoFisher Q32854) kits.

### RNA-SEQ: sequencing

RNA-Sequencing was performed by the High-Throughput DNA Sequencing Core Facility at Georgia Tech. Samples were run on an Illumina HiSeq. 2500 with single-ended, 100 base-pair reads using a cBOT to split lanes before sequencing.

### RNA-SEQ: post-sequencing quality control

Data were processed using the FASTQC Toolkit (v2.0.0, Illumina Basespace). Minimum read length was set to 32. Adapter, base, quality, and Poly-A/T trimming were performed according to previously published methods^64–66^.

### RNA-SEQ: sequence alignment and differential expression

Transcript-level sequence alignment was done using the pseudo-alignment tool, Kallisto (0.43.0)^67^ and the GENCODE v24 transcriptome for Homo sapiens (www.gencodegenes.org)^68^ using 100 bootstrap samples, single-end mode, with the average fragment length and standard deviation obtained for each sample during quality control. Differential expression was performed using Sleuth (v 0.28.1)^69^ in R (v 3.2.1, R Foundation for Statistical Computing).

### RNA-SEQ: pathway over-representation analysis

The differentially expressed transcripts were pre-filtered to exclude transcripts with an absolute fold change of less than 2-fold. For each condition-pair, over-representation analysis was performed using g:profiler version: r1732_e89_eg36 (http://biit.cs.ut.ee/gprofiler)^70^. The Homo sapiens dataset was used and the search included Gene Ontology, Kyoto Encyclopedia of Genes and Genomes (KEGG), CORUM, Reactome, and Regulatory Motif databases and the built-in g:SCS threshold was used to correct p-values for significance cutoff. Default settings were used. QuickGo (https://www.ebi.ac.uk/QuickGO) web service was used for obtaining additional gene ontology information for visualization with Python (v2.7.x, Python Software Foundation, Anaconda Distribution).

### RNA-SEQ: pathway enrichment analysis

Gene Set Enrichment Analysis (GSEA) (v3.0 build: 0160, http://www.broadinstitute.org/gsea)^71^ was used to perform pathway enrichment analysis. Pre-ranked analysis was performed against the Molecular Signatures Database gene sets for: curated data (c2.all.v6.0), gene ontology (c5.all.v6.0), and oncogenic signatures (c6.all.v6.0) gene sets. For the ranking scheme, we used the negative-log of the multiple-hypothesis-corrected p-value (obtained via differential expression analysis in Sleuth) and signed according to the fold-change direction. The transcript with the lowest p-value was selected in cases of gene symbol collision. Default settings were used except the maximum and minimum pathway size exclusion criteria were set to 1,000 and 5, respectively. Significance criteria was p < 0.05 and the GSEA tool recommended false-discovery rate of 0.25.

### Pharmacological inhibitors

Inhibitors used (listed in Table 2) were obtained from SelleckChem, with the exception of Isoginkgetin, which was purchased from Sigma-Aldrich. Doses were selected based on literature and experimental data provided by SelleckChem (see Supplementary Table S4). Inhibitors were solubilized in dimethyl sulfoxide (Sigma-Aldrich, D2650) according to manufacturer instructions and stored in high-concentration aliquots at −80 °C prior to making working dilutions with cell culture media, which were stored at −20 °C for no more than one month prior to experiments. Working solutions were thawed for use immediately beforehand at room temperature.

U87 mg or DAOY spheroidal aggregates were plated in electrotaxis chambers and dcEFs were applied as described above. However, for conditions with inhibitors, appropriately concentrated solutions were added immediately before Matrigel was added to the cell-aggregates/media solution. Cells were again given 24 h in Matrigel (and inhibitor, if specified) before application of dcEF. Analysis of spheroidal aggregates was performed as described above.

### Graphing and Statistics

All graphs and statistics analysis were done with Python (v2.7.x, Python Software Foundation, Anaconda Distribution) or Prism 7 (Graphpad Inc.) unless otherwise specified. The statistical methods used are reported for each result, in place. An α of 0.05 was used unless otherwise specified. Correlations were calculated using pearsonr from scipy.stats.

### Flow cytometry

For ErbB2 cell surface expression, U87 mg cells were plated as 2D dispersed, 3D dispersed, and as spheroidal aggregates with and without Matrigel. Cells and aggregates were trypsinized and stained in phosphate-buffered saline (Corning 21-040-CV) with 10% fetal bovine serum (Gemini 900-108) and 0.09% sodium azide (Sigma-Aldrich, S2002). ErbB2 expression was measured using 1:20 dilutions of APC/Fire 750 anti- human CD340 (BioLegend 324421) and APC/Fire 750 mouse IgG1, κ isotype (BioLegend 400195) as negative control. MCF-7 cells, known to have elevated ErbB2 expression, plated as 2D dispersed were used as positive control for CD340. Cells were analyzed on a Novocyte 2060 flow cytometer. All flow cytometry data analysis was performed using FlowJo vX.07.

### Computer Code Availability Statement

Any python data wrangling code used in this study is available from the corresponding author upon reasonable request. All other code used is cited in the methods and was publicly available at time of manuscript submission.

## Supplementary information

Supplementary Information

Dataset 1

Dataset 2

## Data Availability

Raw RNA-SEQ data and consolidated Sleuth differential expression output has been uploaded to the NCBI Gene Expression Omnibus (GEO accession: GSE115509). Any additional data that support this manuscript not provided directly with the manuscript are available from the corresponding author upon reasonable request.
