# Supplementary material for: Electrotaxis of Glioblastoma and Medulloblastoma Spheroidal Aggregates
Source: Sci Rep. 2019 Mar 29;9:5309. doi: 10.1038/s41598-019-41505-6 (PMC6441013; doi:10.1038/s41598-019-41505-6)
Supplement: Supplementary file 1 — Supplementary Information [file 41598_2019_41505_MOESM1_ESM.docx]

**SUPPLEMENTARY INFORMATION**

Electrotaxis of Glioblastoma and Medulloblastoma Spheroidal Aggregates

Johnathan G. Lyon^1,2*^, Sheridan L. Carroll^1^, Nassir Mokarram^1^, Ravi V. Bellamkonda^1*^

^1^Department of Biomedical Engineering, Pratt School of Engineering, Duke University, 101 Science Drive, Durham, NC 27705, USA

^2^Wallace H. Coulter Department of Biomedical Engineering, Georgia Institute of Technology & Emory School of Medicine, UA Whitaker Building, 313 Ferst Drive, Atlanta, GA 30332, USA

*Corresponding Authors: j.lyon@duke.edu, ravi@duke.edu


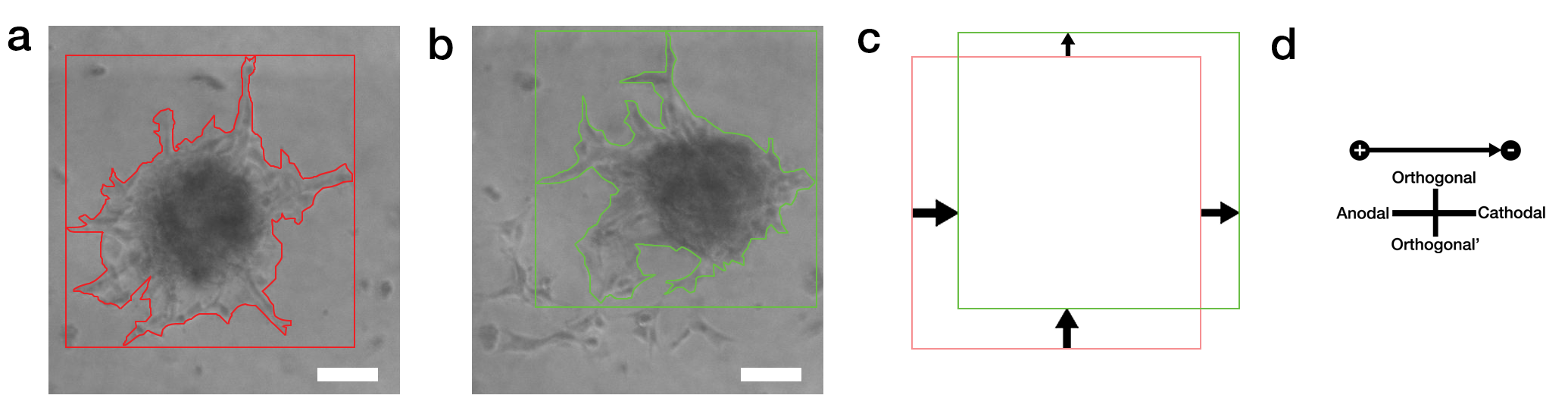


Supplementary Figure S1. Definition of frontier bounding boxes. Frontiers are defined using the maximal extent of each spheroidal aggregate (a & b) in one of four directions as defined in (d). The signed magnitude of outward change (c) in frontier from one time point to the next is used to report change in the aggregates. Scale: 100 μm.

**
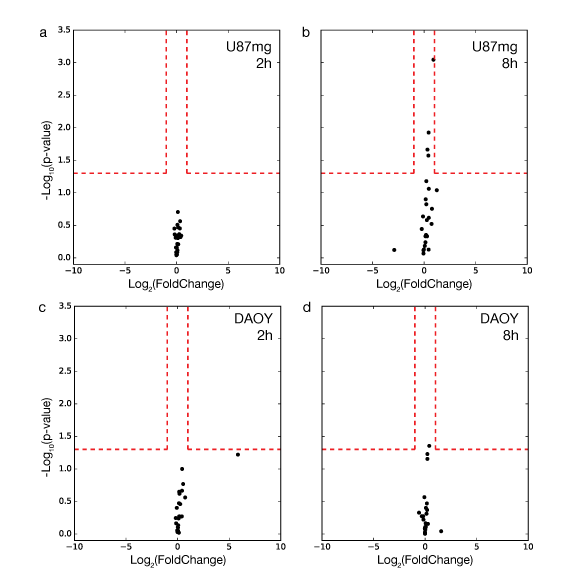
**

Supplementary Figure S2. Volcano plots for qRT-PCR data from electrotaxis candidate genes before multiple hypothesis correction. (a & b) U87mg, or (c & d) DAOY cells were analyzed for gene expression of 25 candidate electrotaxis genes. Shown are the p-values and fold-changes for these genes comparing the expression at (a & c) 2h, or (b & d) 8h after dcEF onset, relative to controls at 0h without dcEF. Significance was determined as any gene with p<0.05 and a Log_2_(fold-change) of greater than 1 or less than -1 (n=4 for all conditions except DAOY 2h, which was n=3—sample dropped during to quality control). No genes were found to be significantly, differentially expression for these conditions.


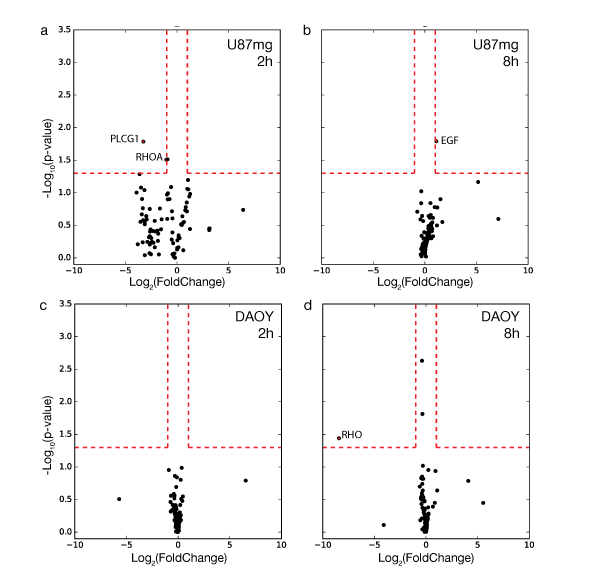


Supplementary Figure S3. Volcano plots for qRT-PCR data from electrotaxis motility genes before multiple hypothesis correction. (a & b) U87mg, or (c & d) DAOY cells were analyzed for gene expression of 25 candidate electrotaxis genes. Shown are the p-values and fold-changes for these genes comparing the expression at (a & c) 2h, or (b & d) 8h after dcEF onset, relative to controls at 0h without dcEF. Significance was determined as any gene with p<0.05 and a Log_2_(fold-change) of greater than 1 or less than -1 (n=4 for all conditions except DAOY 2h, which was n=3—sample dropped during to quality control). While PLCG1, RHOA, RHO and EGF were found to be differentially-expressed under these criteria, after multiple-hypothesis correction was performed on the initial p-values, no genes were found to be significantly, differentially expressed.

**
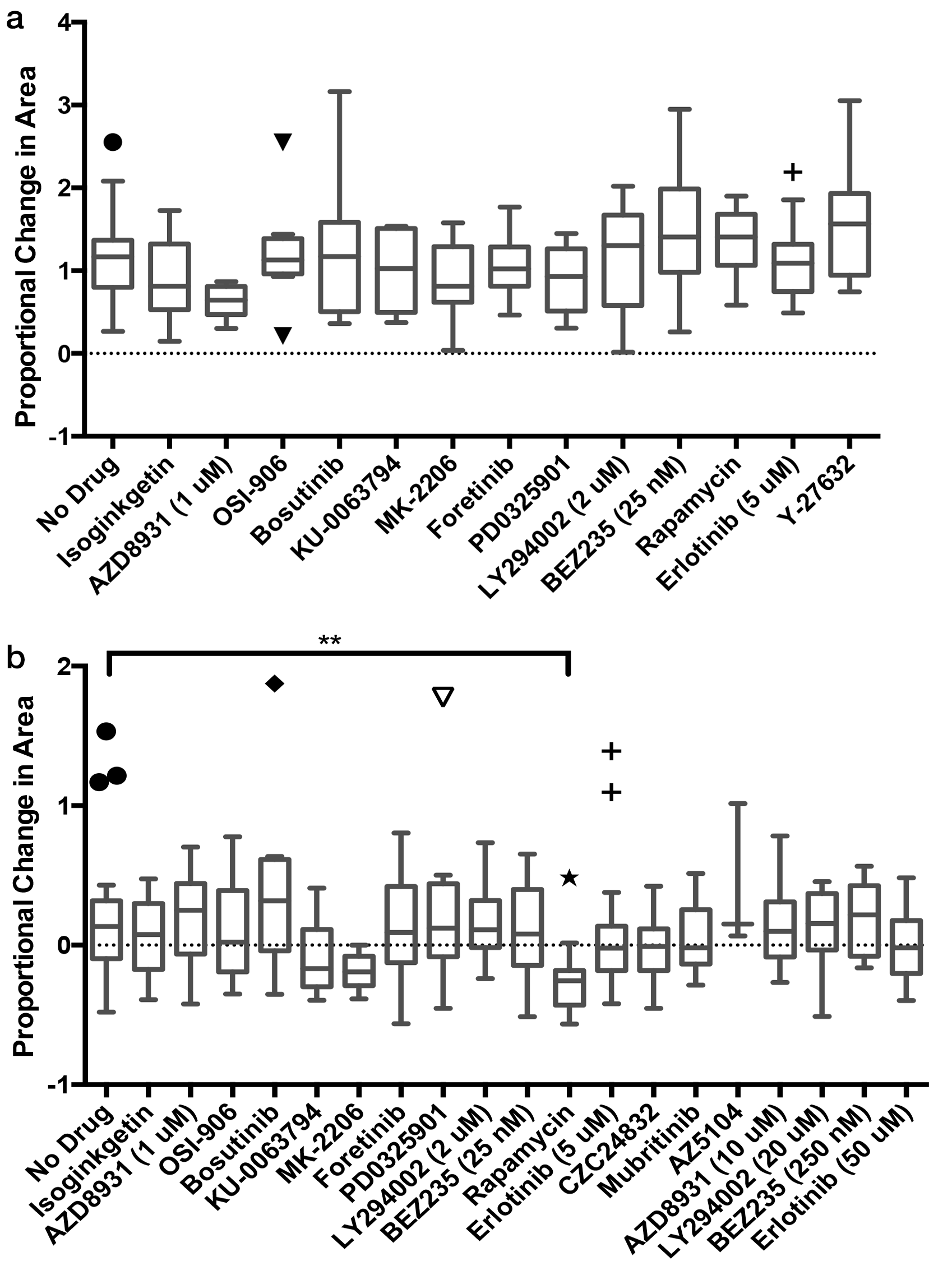
**

Supplementary Figure S4. Proportional change in spheroid bounding box area after exposure to inhibitors. Samples without dcEF shown for (a) DAOY and (b) U87mg cells for the period 24h to 48h after exposure to inhibitor. **p=0.0039. Mean±Tukey box plots shown

**
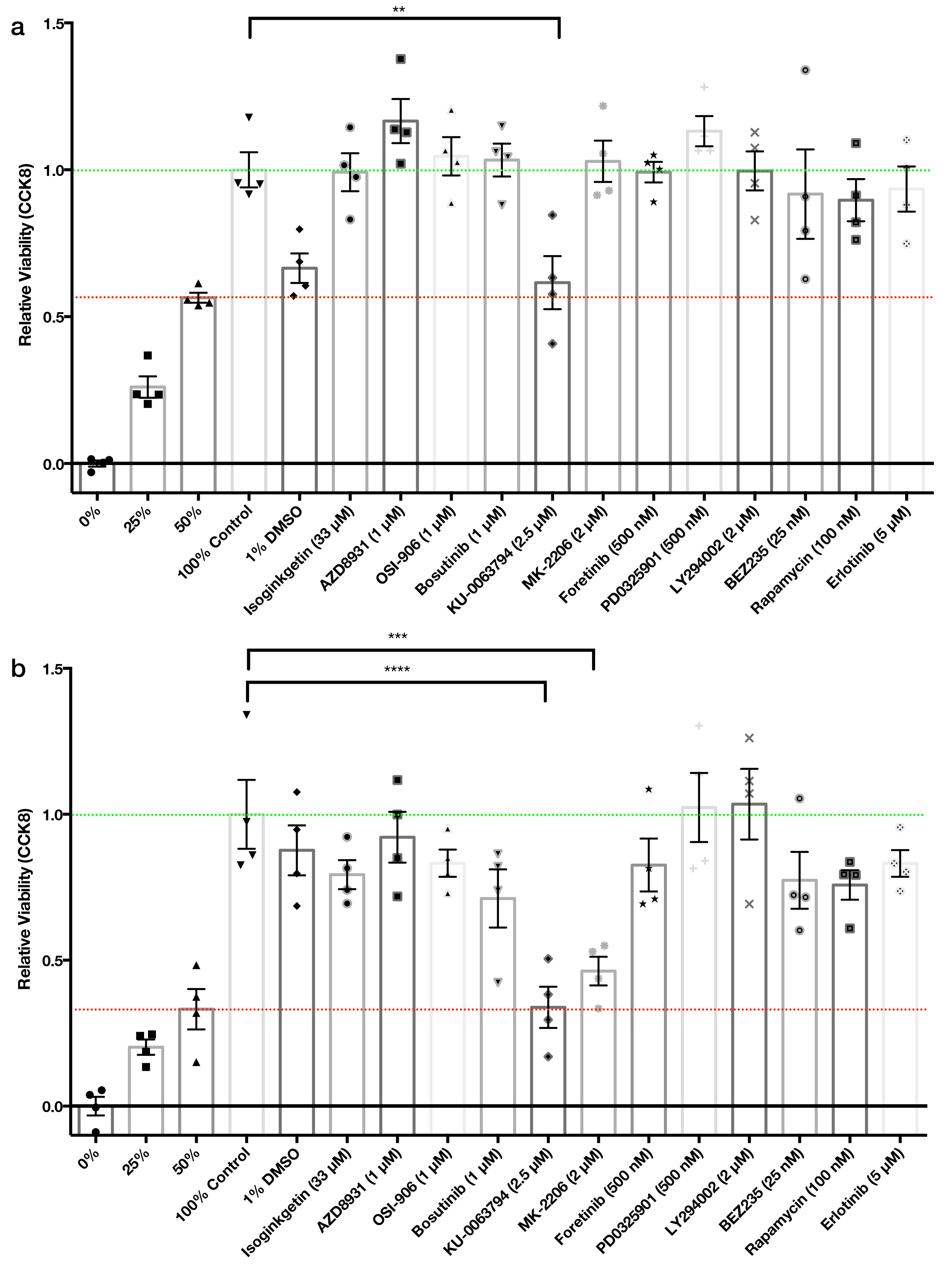
**

Supplementary Figure S5. Viability Assessment with Cell-Counting Kit 8 Assay after addition of inhibitors. Viability after 24h exposure to various inhibitory compounds for (a) U87mg and (b) DAOY cells (n=4 for all conditions). **p=0.0032, ***p=0.0002, ****p<0.0001. 100% control represents 5000 cells initially plated, 50% 2500 cells, etc. 50%, 25%, and 0% were also significantly different than 100% controls, and 1% DMSO control was only significantly different for U87mg cells. Mean±SEM shown.

**
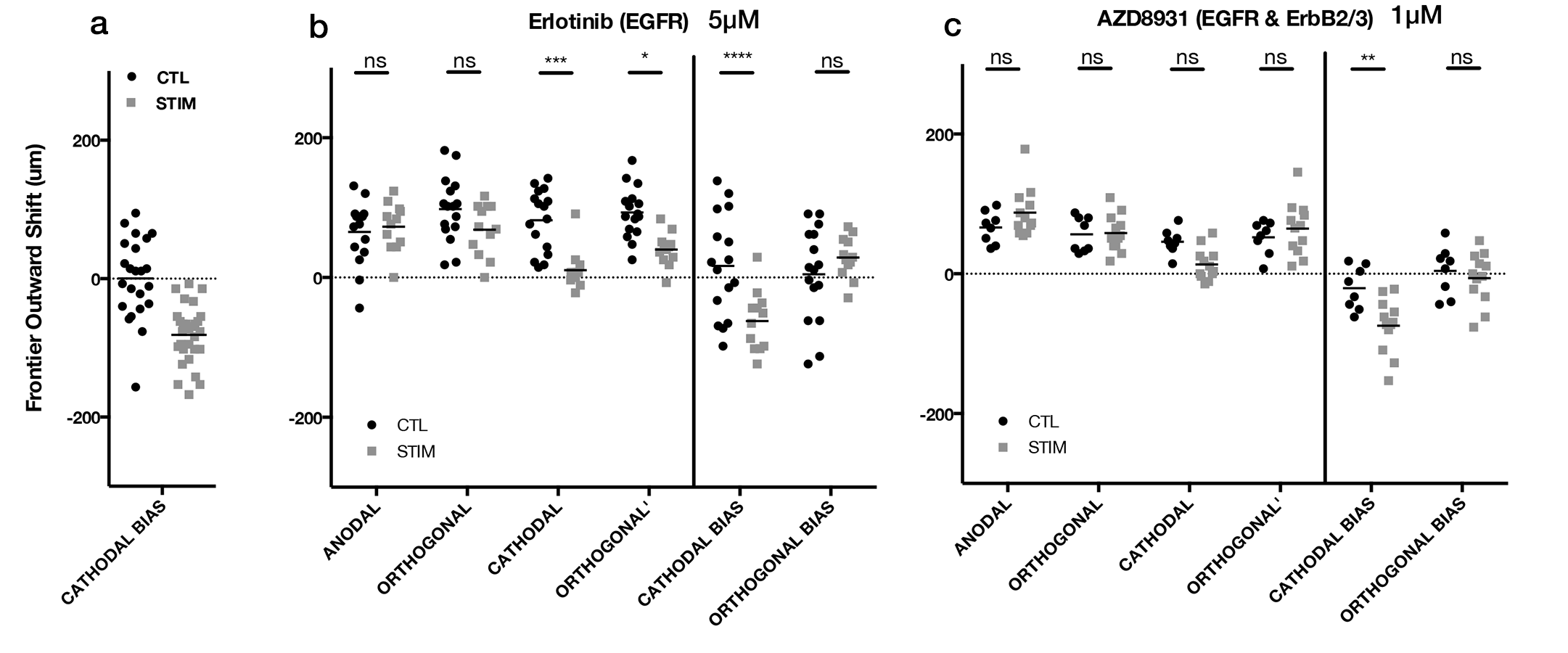
**

Supplementary Figure S6. Effect of ErbB inhibitors on DAOY spheroidal aggregates undergoing electrotaxis. 24h hours after dcEF (stim) or controls without dcEF (CTL) (a) DAOY data without inhibitor compounds, redisplayed from Figure 3b. (b) Erlotinib (5 μM) ***p=0.0004, *p=0.0127, ****p<0.0001 (CTL, n=16; STIM, n=12); (c) AZD6931 (1 μM) **p=0.0014 (CTL, n=8; STIM, n=12); ns=not significant; Two-way ANOVA, Holm-Sidak post-hoc.

**
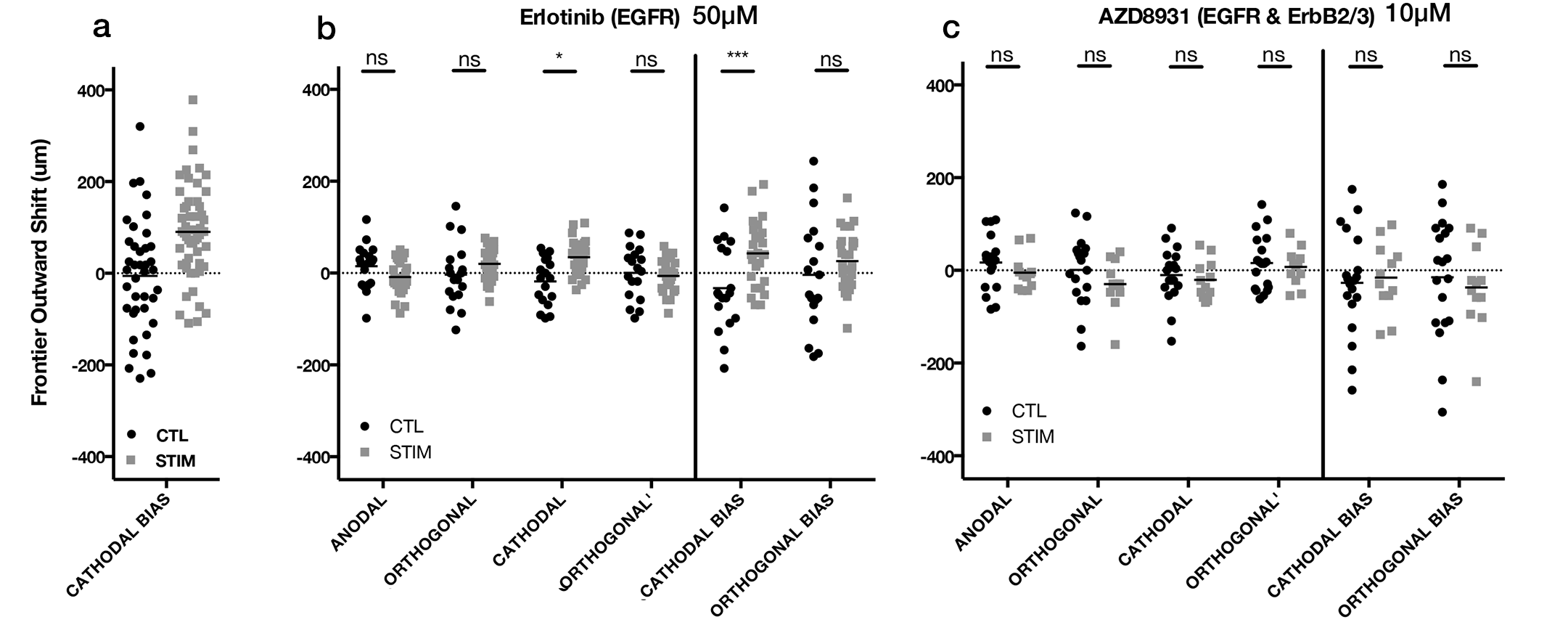
**

Supplementary Figure S7. Effect of ErbB inhibitors at increased dosage on U87mg spheroidal aggregates undergoing electrotaxis. 24h hours after dcEF (stim) or controls without dcEF (CTL) (a) U87mg data without inhibitor compounds, redisplayed from Figure 2d. (b) Erlotinib (50 μM) *p=0.0278, ***p=0.0004 (CTL, n=18; STIM, n=27); (c) AZD6931 (10 μM) (CTL, n=19; STIM, n=12); ns=not significant; Two-way ANOVA, Holm-Sidak post-hoc.

**
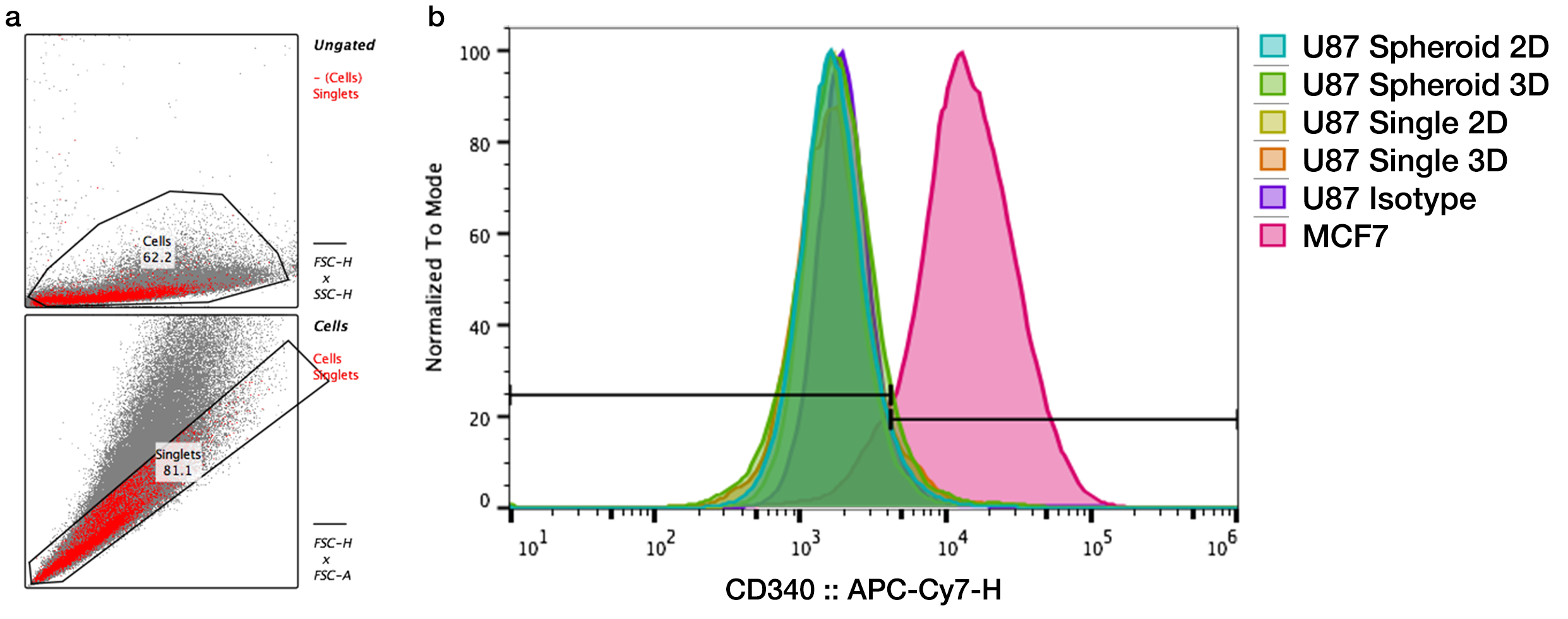
**

Supplementary Figure S8. Flow cytometry data for ErbB2 expression for different plating confirmations of U87mg cells. U87mg cells were plated onto either 2D culture plastic or embedded in 3D Matrigel as either single, dispersed cells, or as spheroidal aggregates. After 24h culture in those conditions, disassociated cells were stained for CD340 (ErbB2 marker) with APC/Fire 750 conjugated antibodies. (a) Gate history showing selection of cells and singlets. (b) Histogram of APC/Fire 750 intensity. ErbB2+ MCF7 cells serve as positive control.

**
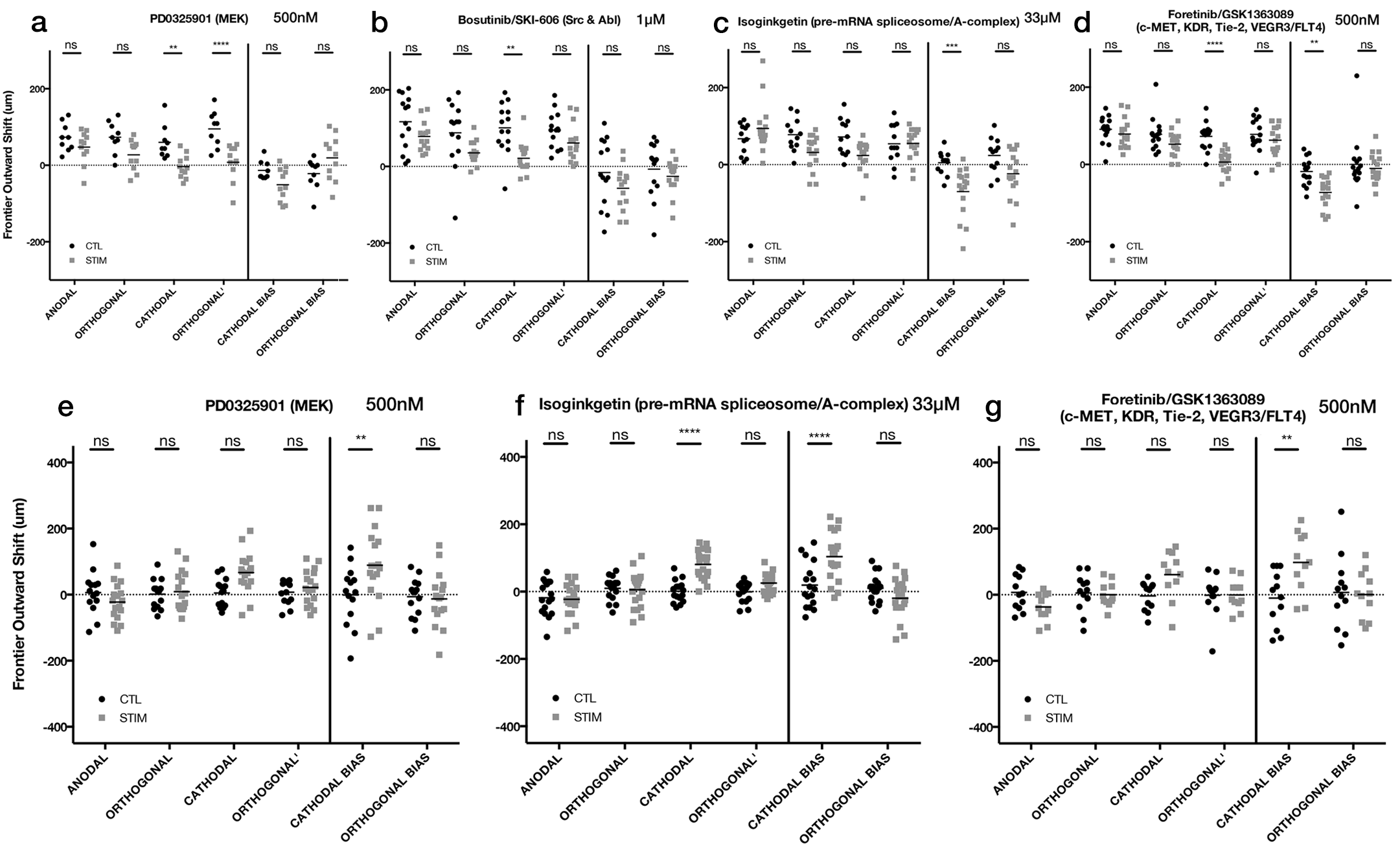
**

Supplementary Figure S9. Effect of Additional inhibitors on DAOY (a-d) and U87mg (e-g) spheroidal aggregates undergoing electrotaxis. 24h hours after dcEF (stim) or controls without dcEF (CTL); (a) PD0325901 (500 nM) **p=0.0058, ****p<0.0001 (CTL, n=9; STIM, n=11); (b) Bosutinib (1 μM) **p=0.0035 (CTL, n=14; STIM, n=14); (c) Isoginkgetin (33 μM) ***p=0.0006 (CTL, n=12; STIM, n=16); (d) Foretinib (500 nM) **p=0.0016, ****p<0.0001 (CTL, n=15; STIM, n=16); (e) PD0325901 (500 nM) **p=0.0015 (CTL, n=14; STIM, n=17); (f) Isoginkgetin (33 μM) ****p<0.0001 (CTL, n=17; STIM, n=20); (g) Foretinib (500 nM) **p=0.0013 (CTL, n=12; STIM, n=11); ns=not significant; Two-way ANOVA, Holm-Sidak post-hoc.

Supplementary Table S1. qRT-PCR primers for candidate electrotaxis genes

| **GENE** | **NCBI Refseq** | **Forward Primer** | **Reverse Primer** |
| --- | --- | --- | --- |
| AKT1 | NM_005163.2 | CACACACTCACCGAGAACC | TCGTGGGTCTGGAAAGAGTA |
| CCL2 | NM_002982.3 | TAGCAGCCACCTTCATTCCC | CCTCTGCACTGAGATCTTCCTA |
| CDC42 | NM_001791.3 | GGTGGAGAACCATATACTCTTGG | GGATAACTCAGCGGTCGTAAT |
| EGFR | NM_005228.3 | GCAGTGACTTTCTCAGCAACA | TTGGGACAGCTTGGATCACA |
| FGF2 | NM_002006.4 | TGCTAACCGTTACCTGGCTA | AAGAAACACTCATCCGTAACACA |
| FLT1 | NM_001159920.1 | TAGCTGGCAAGCGGTCTTAC | GCAGATTTCTCAGTCGCAGGTA |
| FRK | NM_002031.2 | TGTCAAGCTGGGGAAACCA | TCCCATTGGTCCACGGTTTTA |
| FYN | NM_153047.1 | GAGCCCATCTACATCGTCAC | TTCAGAGCTCTTCCTTCTCCA |
| IPCEF1 | NM_001130700.1 | TCAGAAGCCCAGGAGGAAA | GCATGGCCCAGATCTTTACA |
| MAPK1 | NM_002745.4 | TTGGTACAGGGCTCCAGAAA | TCTGCCAGAATGCAGCCTA |
| MAPK14 | NM_001315.2 | GGCTCCTGAGATCATGCTGAA | ACAGCTCGGCCATTATGCA |
| MAPK3 | NM_002746.2 | GAAGATCAGCCCCTTCGAACA | GCAGCAGGATCTGGATCTCC |
| MAPK8 | NM_002750.2 | TCTCCAACACCCGTACATCA | CCCTTTCATCTAACTGCTTGTCA |
| MMP1 | NM_001145938.1 | CACCTTCAGTGGTGATGTTCA | GCTGGACAGGATTTTGGGAA |
| MMP9 | NM_004994.2 | AGTGGCACCACCACAACA | GCAAAGGCGTCGTCAATCA |
| PIK3CA | NM_006218.2 | CTGCAGTTCAACAGCCACAC | ACAGGTCAATGGCTGCATCA |
| PRKACA | NM_002730.3 | AAGGAGACCGGGAACCACTA | AGGGTGTGTTCGATCTGTTTCA |
| PRKCA | NM_002737.2 | ACCATCCGCTCCACACTAAA | AGTCGTCGGTCTTTGTCTGAA |
| PTEN | NM_000314.4 | CCAGACATGACAGCCATCA | AGTCTTTCTGCAGGAAATCCC |
| RAC1 | NM_018890.3 | TCACCTATCCGCAGGGTCTA | GCCGAGCACTCCAGGTATTTTA |
| RHOA | NM_001664.2 | GTGCCCACAGTGTTTGAGAA | TGTGTCCCACAAAGCCAAC |
| SCN1B | NM_000314.4 | TTCACCGAGTGGACCTTCC | TCCAGCTGCAACACCTCA |
| SGK1 | NM_001037.4 | CTTGGGCTACCTGCATTCAC | TGTGTCCCTGTGAATCTAGCA |
| SOD2 | NM_001024465.1 | AGGAACGGGGACACTTACAA | TCAATCCCCAGCAGTGGAATA |
| VEGFC | NM_005429.2 | GCCAACCTCAACTCAAGGAC | GCATGCATTGAGTCTTTCTCCA |
| **HOUSEKEEPING GENES** | |  |  |
| ACTB | NM_001101.3 | CCAACCGCGAGAAGATGAC | TAGCACAGCCTGGATAGCAA |
| GAPDH | NM_002046.4 | GAACGGGAAGCTTGTCATCAA | ATCGCCCCACTTGATTTTGG |
| IPO8 | NM_001190995.1 | TTCAGTGCAAAGGAAGGGGAA | ACCCCTCGAGTTAATCTCTCCA |
| RPL13A | NM_012423.3 | GAGGCCCCTACCACTTCC | GCCGTCAAACACCTTGAGAC |
| SDHA | NM_004168.2 | ACATCGGAACTGCGACTCA | TTCTTGCAACACGCTTCCC |
| TBP | NM_001172085.1 | TGCCCGAAACGCCGAATATA | CGTGGTTCGTGGCTCTCTTA |

Supplementary Table S2. Motility qRT-PCR Genes grouped by functional category (primer sequences not provided by SABiosciences)

| **FUNCTION** | **GENES** |
| --- | --- |
| **Chemotaxis** | FGF2, ITGB2, MAPK1, MYH10, MYH9, PLAUR, PLD1, PRKCA, RAC2, TGFB1,VEGFA, WASF2, WIPF1 |
| **Receptors** | EGFR, IGF1R, ITGA4, ITGB1, ITGB2, ITGB3, MET, PLAUR, RHO |
| **Growth Factors** | CSF1, EGF, FGF2, HGF, IGF1, TGFB1, VEGFA |
| **Rho Family GTPases** |  |
| Rho Signaling | ACTR2, ACTR3, ARHGDIA, LIMK1, MSN, MYL9, MYLK, PLCG1, PLD1, PRKCA, PTEN, PTPN1,RHO, RHOA, RHOB, RHOC, RND3, ROCK1, VIM |
| Rac Signaling | ACTR2, ACTR3, BAIAP2, CFL1, CRK, PAK1, PAK4, PLD1, PRKCA, RAC1, RAC2, STAT3, WASF1,WASF2, WAS |
| CDC42 Signaling | ACTR2, ACTR3, CDC42, PFN1, WASF1, WASF2, WASL |
| **Adhesion** |  |
| Cell-Cell Adhesion | DPP4, EGFR, EZR, ITGA4, ITGB1, ITGB2, MSN, MYH9, ROCK1, TGFB1 |
| Cell-Matrix Adhesion | ACTN1, ACTN3, CSF1 (MCSF), ILK, ITGB1, ITGB2, ITGB3, MMP14, PTEN, PTK2B, PXN, RASA1, RHOA |
| Focal Adhesion | ACTN1, ACTN3, ARHGEF7, BCAR1, CAPN1, CAPN2, CAV1, ENAH, ILK, ITGB1, MYL9, PTK2,PTK2B, PXN, TLN1, VASP, VCL |
| Leukocyte Adhesion & Rolling | EZR, ITGA4, ITGB1, ITGB2, MSN, ROCK1 |
| **Integrin-Mediated Signaling** | BCAR1, ILK, ITGA4, ITGB1, ITGB2, ITGB3, MYH9, PTK2 |
| **Cellular Projections** |  |
| Filopodia | BAIAP2, CDC42, DIAPH1, EGFR, ENAH, EZR, MSN, RDX, SVIL, VASP |
| Lammelipodia | CTTN, DPP4, EGFR, ENAH, FAP, PIK3CA, PLD1, PTK2, PXN, RDX, SVIL, VASP, VCL,WASF1, WASF2, WASL |
| Stress Fibers | ACTN4, DIAPH1, MYH10, MYH9, RHOA, RHOB, RHOC |
| Membrane Blebs | ACTN1, ACTN3, ACTN4, EZR, MYH10, MYH9, MYLK, RND3, ROCK1 |
| Invasive Projections | ACTR2, ACTR3, ARF6, CDC42, CFL1, CTTN, DPP4, EGF, EZR, FAP, MMP14, MMP2, MMP9, MSN, MYH9, PLAUR, RAC2, RASA1, SH3PXD2A, SRC, SVIL, TGFB1,VEGFA, WASL, WIPF1 |
| Growth Cones | ARHGEF7, CDC42, CFL1, PTK2B |
| Membrane Ruffles | ACTR2, ACTR3, ARF6, BAIAP2, BCAR1, CTTN, DIAPH1, EZR, ITGB1, MYH9, RAC1, RAC2,RDX, RHOA, TLN1, WASF2 |
| **Cell Polarity** | CDC42, CFL1, EZR, IGF1R, ILK, MYH9 |
| **Proteolysis** | AKT1, CAPN1, CAPN2, DPP4, FAP, HGF, MMP14, MMP2, MMP9, MYH9, PLAUR, TIMP2 |

Supplementary Table S3. TRANSFAC transcription factor motifs significantly over-represented

| **U87 only**  EHF  ELK1  ETS1  ETV5  ETV7  FOXC1  PSF | **DAOY only**  AHR  BARHL2  c-ETS1  CDX2  CHD2  Churchill  DP2  E2F3  ELF2  ELK4  ER71  ERG  ERM  FOXJ2  GABPA  GR  HMX3  HNF1B  HOXA13  HOXA9  LHX3  MSX1  NRF1  OCT4  PAX6  PET1  REX1  RNF96  SP1  SP3  TEF3  TEL1  ZBTB7C | **Both U87 & DAOY**  CPBP  DP1  E2F  E2F1  E2F4  EGR1  ELF1  ELF4  ER81  ETF  FLI1  RB  SP2  SP6  ZF5 |
| --- | --- | --- |

Supplementary Table S4. Literature and Data used to determine experimental doses for pharmacological inhibitors. Note, when available, prior data specific for U87mg or DAOY cells was used. *data from Sigma-Aldrich; †reported full target inhibition; ‡50 nM for HER2 IC50; NR: not reported

| **Drug** | **in vitro IC50 (from SelleckChem)** | **Previous in vitro use** | **Reference** |
| --- | --- | --- | --- |
| Isoginkgetin | 30 uM* | 33um in HEK | [58] |
| AZD8931 | 4 nM | 0.001-10 uM in various | [73] |
| OSI-906 | 1 uM† | 0.02-0.8 uM in various | [74] |
| Bosutinib | 100 nM | 250 nM in MDA | [75] |
| KU-0063794 | 30 nM | 5 uM in U373 | [76] |
| MK-2206 | NR | 1-10uM in U87 | [77] |
| Foretinib | 0.4-165 nM | .5-2.5uM in DAOY | [78] |
| PD0325901 | 2-123 nM | 0.1-1uM in various | [79] |
| LY294002 | 0.38-45 uM | 20 uM in U87/U251 | [23] |
| BEZ235 | 75 nM | 0.001-0.1uM in U87 | [80] |
| Rapamycin | 0.1nM-1uM | 100 nM in U87 | [81] |
| Erlotinib | 20 nM-20uM | 1-10uM in U87 | [82] |
| CZC24832 | 1.5uM | 1 uM in Multiple myeloma cells | [83] |
| Mubritinib | 0.1uM-25uM | 0.25-1 uM in AML cells | [84] |
| Y-27632 | 0.3-1uM | 10 uM in DAOY | [85] |
| AZ5104 | 2.6-80 nM‡ | 3-80 nM in Lung Cancers | [86] |
|  |  |  |  |
|  |  | |  |
|  |  | |  |
|  |  | |  |
|  |  |  |  |

**REFERENCES**

1. Hickinson, D. M., Klinowska, T., Speake, G., Vincent, J., Trigwell, C., Anderton, J., Beck, S., Marshall, G., Davenport, S., Callis, R. & Mills, E. AZD8931, an equipotent, reversible inhibitor of signaling by epidermal growth factor receptor, ERBB2 (HER2), and ERBB3: a unique agent for simultaneous ERBB receptor blockade in cancer. *Clinical Cancer Research* **16**(4), 1159-1169 (2010).
2. Mulvihill, M. J., Cooke, A., Rosenfeld-Franklin, M., Buck, E., Foreman, K., Landfair, D., O’Connor, M., Pirritt, C., Sun, Y., Yao, Y. & Arnold, L. D. Discovery of OSI-906: a selective and orally efficacious dual inhibitor of the IGF-1 receptor and insulin receptor. *Future medicinal chemistry* **1**(6), 1153-1171 (2009).
3. Vultur, A., Buettner, R., Kowolik, C., Liang, W., Smith, D., Boschelli, F., & Jove, R. SKI-606 (bosutinib), a novel Src kinase inhibitor, suppresses migration and invasion of human breast cancer cells. *Molecular Cancer Therapeutics* **7**(5), 1185-1194 (2008).
4. Fan, Q. W., Cheng, C., Hackett, C., Feldman, M., Houseman, B. T., Nicolaides, T., ... & Shokat, K. M. Akt and autophagy cooperate to promote survival of drug-resistant glioma. *Science signaling* **3**(147), ra81 (2010).
5. Jin, R., Nakada, M., Teng, L., Furuta, T., Sabit, H., Hayashi, Y., Demuth, T., Hirao, A., Sato, H., Zhao, G. & Hamada, J. I. Combination therapy using Notch and Akt inhibitors is effective for suppressing invasion but not proliferation in glioma cells. *Neuroscience letters* **534**, 316-321 (2013).
6. Faria, C. C., Golbourn, B. J., Dubuc, A. M., Remke, M., Diaz, R. J., Agnihotri, S., Luck, A., Sabha, N., Olsen, S., Wu, X. & Garzia, L. Foretinib is effective therapy for metastatic sonic hedgehog medulloblastoma. *Cancer research* **75**(1), 134-146 (2015).
7. See, W. L., Tan, I. L., Mukherjee, J., Nicolaides, T., & Pieper, R. O. Sensitivity of glioblastomas to clinically available MEK inhibitors is defined by neurofibromin 1 deficiency. *Cancer research* **72**(13), 3350-3359 (2012).
8. Liu, T. J., Koul, D., LaFortune, T., Tiao, N., Shen, R. J., Maira, S. M., Garcia-Echevrria, C. & Yung, W. A. NVP-BEZ235, a novel dual phosphatidylinositol 3-kinase/mammalian target of rapamycin inhibitor, elicits multifaceted antitumor activities in human gliomas. *Molecular cancer therapeutics* **8**(8), 2204-2210 (2009).
9. Takeuchi, H., Kondo, Y., Fujiwara, K., Kanzawa, T., Aoki, H., Mills, G. B., & Kondo, S. Synergistic augmentation of rapamycin-induced autophagy in malignant glioma cells by phosphatidylinositol 3-kinase/protein kinase B inhibitors. *Cancer research* **65**(8), 3336-3346 (2005).
10. Fan, Q. W., Cheng, C. K., Nicolaides, T. P., Hackett, C. S., Knight, Z. A., Shokat, K. M., & Weiss, W. A. A dual phosphoinositide-3-kinase α/mTOR inhibitor cooperates with blockade of epidermal growth factor receptor in PTEN-mutant glioma. *Cancer research* **67**(17), 7960-7965 (2007).
11. Piddock, R. E., Loughran, N., Marlein, C. R., Robinson, S. D., Edwards, D. R., Yu, S., Pillinger, G. E., Zhou, Z., Zaitseva, L., Auger, M. J. & Rushworth, S. A. PI3K-[delta] and PI3K-[gamma] isoforms have distinct functions in regulating pro-tumoural signalling in the multiple myeloma microenvironment. *Blood cancer journal* **7**(3), e539 (2017).
12. Ufkin, M. L., Peterson, S., Yang, X., Driscoll, H., Duarte, C., & Sathyanarayana, P. miR-125a regulates cell cycle, proliferation, and apoptosis by targeting the ErbB pathway in acute myeloid leukemia. *Leukemia research* **38**(3), 402-410 (2014).
13. Coniglio, S. J., Zavarella, S., & Symons, M. H. Pak1 and Pak2 mediate tumor cell invasion through distinct signaling mechanisms. *Molecular and cellular biology* **28**(12), 4162-4172 (2008).
14. Cross, D. A., Ashton, S. E., Ghiorghiu, S., Eberlein, C., Nebhan, C. A., Spitzler, P. J., Orme, J. P., Finlay, M. R. V., Ward, R. A., Mellor, M. J. & Hughes, G. AZD9291, an irreversible EGFR TKI, overcomes T790M-mediated resistance to EGFR inhibitors in lung cancer. *Cancer discovery* **4**(9), 1046-1061 (2014).
